# Impact of CaNa_2_EDTA fortification on growth, antioxidant activity and stress-related gene expression in tilapia (*Oreochromis niloticus*) at different stocking densities paradigms

**DOI:** 10.1371/journal.pone.0316629

**Published:** 2025-01-06

**Authors:** Wajeeha Komal, Shafaq Fatima, Qandeel Minahal, Razia Liaqat, Asma Abdul Latif, Aya S. Hussain

**Affiliations:** 1 Department of Zoology, Faculty of Natural Sciences, Lahore College for Women University, Lahore, Punjab, Pakistan; 2 Department of Forestry and Natural Resources, Purdue University, West Lafayette, Indiana, United States of America; 3 Zoology Department, Faculty of Science, Suez University, Suez, Egypt; Tanta University Faculty of Agriculture, EGYPT

## Abstract

Increasing aquaculture production requires high-density farming, which induces stress, necessitating supplements to mitigate its effects and ensure fish health. The aim of this study was to examine how CaNa_2_EDTA (EDTA) affects the growth, immune response and antioxidant activity in Nile tilapia (*Oreochromis niloticus*). The fish were raised at three different stocking densities: low (LD = 2.00 kg/m^3^), medium (MD = 3.50 kg/m^3^), and high (HD = 5.00 kg/m^3^). Each density group was fed with one of four levels of EDTA supplementation (E0 = 0 g/kg, E1 = 5 g/kg, E2 = 10 g/kg, and E3 = 15 g/kg) for 60 days. Each diet was tested in triplicate (n = 66 fish per replicate in LD, 116 per replicate in MD, and 166 per replicate in HD). After 60 days, the results of this study declared that LD group showed better growth than the MD and HD groups, and among all groups, those fed the E1 diet grew better than those on other diets. The study found significant changes in the chemical composition of the fish and the activity of digestive enzymes across all treatments. Antioxidant enzyme levels and cortisol were higher in the HD group compared to the LD and MD groups. However, fish in the HD group fed the E1 diet had the lowest levels of antioxidant enzymes and cortisol. Malondialdehyde levels were higher in the HD group compared to the LD and MD groups, with the lowest levels seen in fish on the E1 diet in the HD group. The expression of Somatostatin-1 did not increase in the MD group compared to the LD and HD groups. The gene expression levels of pro-opiomelanocortin-α and Interleukin 1-β were not significantly affected by either stocking density or EDTA supplementation. In conclusion, EDTA supplementation improved growth and antioxidant response in tilapia, with the best results seen at a dose of 5 g/kg in the high-density group, suggesting that this approach could be beneficial in intensive tilapia farming.

## 1. Introduction

Aquaculture offers a promising solution for future high-quality protein needs in the food industry boasting an annual growth rate of 8% [1]. Enhancing fish growth through dietary supplementation are crucial for sustainable fish farming [2, 3]. Rising global fish demands drive aquaculture growth, with tilapia production reaching seven million tonnes in 2020 [1]. Nile tilapia is ideal for aquaculture due to its rapid growth, low-cost diet, and adaptability to high-density systems [4]. The adaptability of Nile tilapia to high-density systems is primarily attributed to genetic improvements, enhancing growth rates, feed efficiency, stress tolerance, and disease resistance [5].

Intensive farming boosts fish yield and investment returns but also increases disease risk and stress due to overcrowding [6]. High stocking density can impact fish growth, which can be measured by growth-related gene expression. Somatostatin-1 (SST-1) regulates energy balance and metabolism, inhibiting growth hormone release, making it a key indicator of growth performance [7–9].

Overcrowding negatively affects physiological responses, as immunosuppression disease resistance by degrading water quality [10–13], high cortisol levels [14, 15], higher mortality [16] causing oxidative stress [14, 17, 18]. This stress elevates cortisol levels via the hypothalamic-pituitary-interrenal (HPI) axis and stimulates corticotropin-releasing hormone production [19]. Impact of stocking density on fish physiology can be evaluated through alkaline phosphatase (ALP), aspartate aminotransferase (AST) levels [20], hemoglobin, red blood cells, albumin, globulin, triglycerides [21] and immune cells [22], blood hematology and biochemistry and glucose levels [23, 24].

Overcrowding induces oxidative stress, resulting in increased reactive oxygen species (ROS) [25] and various forms of cellular damage, including DNA mutations [26]. Fish respond to oxidative stress through stress-related genes such as pro-opiomelanocortin-α, activated via the hypothalamic-pituitary-adrenal (HPA) axis, which releases corticotropin-releasing factor and synthesizes pro-opiomelanocortin (POMC). POMC is cleaved into peptides like α-, β-, and γ-MSH [27]. Adrenocorticotropic hormone from these peptides activates melanocortin-2 receptors, promoting cortisol and corticoid production, which enhances glucose metabolism to help fish cope with stress [28, 29]. Additionally, stress alters immune-related gene expression, notably affecting the proinflammatory cytokine interleukin-1 beta (IL-1β), which regulates the HPA axis and modulates immune response and stress relief [30–32].

Overcrowding deteriorates water quality, marked by low pH, oxygen levels and increased nitrate, ammonia and nitrite concentrations, aggravated by leftover feed [33]. Poor water quality, oxidative stress, and uneaten feed contribute to metal ion production, potentially contaminating fish. Aquaculture feeds containing essential micronutrients like copper, zinc, and iron often lead to heavy metal contamination as uneaten feed decomposes, releasing metals into water [34]. Heavy metals in aquaculture originate from various sources, including agricultural activities [35] and contaminated feed [36] posing risks to ecosystems and human health [37]. The oxidative stress can be alleviated by adding natural or synthetic substances with antioxidant properties like probiotics [38], Vitamin C and oxidized fish oil [39]. Ethylenediamine tetraacetic acid (EDTA) functions as a sequestrant, binding trace minerals to prevent oxidation [40]. Due to its low biodegradability and ability to reduce toxic metal levels, EDTA improves water quality and reduces stress in aquaculture [41, 42]. By inhibiting metal-catalyzed free radical reactions, EDTA mitigates oxidative stress and protects aquatic organisms [43, 44]. Its high affinity for toxic metals like lead and mercury allows for their removal from tissues [45], as EDTA chelates extracellular heavy metals by penetrating cell membranes [46].

This study aims to alleviate the stress associated with high-density fish farming by supplementing fish feed with ethylenediaminetetraacetic acid (EDTA). EDTA acts as a chelating agent, binding to metal ions and reducing their bioavailability, thereby mitigating their potential harm to fish health. By exploring the efficacy of EDTA supplementation, this research seeks to enhance fish welfare, improve growth performance, and ultimately contribute to more sustainable aquaculture practices. This study investigated the efficacy of EDTA in reducing oxidative stress in tilapia and aimed to determine the optimal dose for different stocking densities. The research focused on assessing EDTA impact on growth, antioxidant status, and the expression of key stress and immune-related genes, including POMC-α, IL-1β, and SST-1. By evaluating these factors, the study aimed to explore EDTA’s potential to enhance growth and immunity, particularly in high-density farming conditions, where stress is prevalent. The findings provide insights into improving fish health and productivity in commercial aquaculture.

## 2. Materials and methods

### 2.1. Diet preparation

The feed supplement utilized in this investigation was commercial calcium disodium ethylene diamine tetraacetic acid (EDTA) (Ca (OOCCH2)2NCH2CH2N(CH2COONa)2·aq, Sigma Aldrich, USA; purity >97%). Four different doses of EDTA were used to formulate the treatment diets: E0 (0 g/kg), E1 (5 g/kg), E2 (10 g/kg), and E3 (15 g/kg)—by combining finely powdered components Table 1. At PCSIR Laboratories in Pakistan, a mechanical pellet machine was used to create the 1 mm feed pellets. The feed pellets were dried in ambient air and subsequently stored in sealed bags at a temperature of 4°C. The recommended standard feeding ration for tilapia (*Oreochromis niloticus*) is 2% [47]. The feed utilized in this study contained 30% crude protein, aligning with the tilapia protein requirement range of 25–30%. The dietary requirement for tilapia in context to total energy content (calorie) is 2,500–3,000 kcal/kg and a fiber content ranging between 3% and 7% [47].

**Table 1:** Feed formulation with EDTA supplementation.

Ingredients (%)	E0	E1	E2	E3
Corn meal	28.10	28.10	28.10	28.10
Rice polish	12.10	12.10	12.10	12.10
Wheat bran	9.10	9.10	9.10	9.10
Canola meal	8.10	8.10	8.10	8.10
Soybean meal	36.05	36.05	36.05	36.05
Dicalcium phosphate	3.85	3.85	3.85	3.85
Methionine	0.69	0.69	0.69	0.69
Lysine	1.22	1.22	1.22	1.22
L-Threonine	0.79	0.79	0.79	0.79
EDTA	0.00	0.5	01	1.5
**Chemical composition of feed**				
Moisture (%)	11.45	11.48	11.55	11.59
Crude protein (%)	30.00	30.00	30.00	30.00
Crude fat (%)	7.40	8.00	7.66	7.98
Crude ash (%)	7.98	7.11	7.09	7.16

### 2.2. Experimental design

A 4,200 tilapia (initial weight = 30.00 ± 1.20 g) were brought to the Lahore College for Women University’s Aquaculture Facility from a nearby fish hatchery in Lahore, Pakistan. There were no fish deaths during the transportation. After receiving ethical approval from the Lahore College for Women University Department of Zoology’s Animal Ethics Committee (Approval #: Zoo/LCWU/932), the study got underway. Thirty-six fiberglass tanks, each holding 1 meter^3^ of water, were randomly assigned to the fish. Each tank had its own water source, and all tanks shared a common sump that was treated with UV and biofilters. A week of acclimation preceded the commencement of the experiment for the fish. All fish stayed healthy for the full 60 days of the study. It is generally advised to evaluate the impact of dietary supplements on fish growth over a 60-day period. For this experiment, the intended humane endpoint was 60 days. As explained in section 2.3, fish husbandry and health were closely monitored throughout the experiment to reduce mortality.

In this trial, three distinct stocking density regimes were considered: low density (LD) at 2.00 kg/m^3^, medium density (MD) at 3.50 kg/m^3^, and high density (HD) at 5.00 kg/m^3^. The total number of fish stocked in the LD, MD, and HD treatments was 800, 1,400, and 2,000, respectively. Each density treatment was conducted in triplicate Table 2. Fish in all density treatments (LD, MD, HD) were fed with four different levels of EDTA dietary supplementation i.e. (E0, E1, E2, E3) along with three replicates. Dose of EDTA in each dietary level is given in section 2.1. These four different levels of EDTA were fed to low density treatment (LDE0, LDE1, LDE2, LDE3), medium density treatment (MDE0, MDE1, MDE2, MDE3), and high-density treatment (HDE0, HDE1, HDE2, HDE3). Fish were hand-fed with a daily ration calculated at 4% of the biomass in each replicate. They were fed three times a day throughout the study period. To ensure accurate feeding, the fish in each replicate were randomly weighed every 15 days, and the daily ration was adjusted accordingly.

**Table 2:** Distribution of fish (initial weight = 30.00±1.30g) in experimental design having three stocking densities (LD, MD, HD) and their replicates at four different level of CaNa_2_ EDTA supplementation (E0,E1,E2,E3).

Treatments	Total fish (N)	Stocking density (kg/m^3^)	Number of replicates	Number of fish per replicate	Supplementation levels of EDTA (g/kg)	Distribution of fish in each replicate at four supplementation levels of EDTA
Low Density (LD)	800	2.00	3	266	E0 = 0	LDE0: n = 66
	800	2.00	3	266	E1 = 5	LDE1: n = 66
	800	2.00	3	266	E2 = 10	LDE2: n = 66
	800	2.00	3	266	E3 = 15	LDE3: n = 66
Medium Density (MD)	1400	3.50	3	466	E0 = 0	MDE0: n = 116
	1400	3.50	3	466	E1 = 5	MDE1: n = 116
	1400	3.50	3	466	E2 = 10	MDE2: n = 116
	1400	3.50	3	466	E3 = 15	MDE3: n = 116
High Density (HD)	2000	5.00	3	666	E0 = 0	HDE0: n = 166
	2000	5.00	3	666	E1 = 5	HDE1: n = 166
	2000	5.00	3	666	E2 = 10	HDE2: n = 166
	2000	5.00	3	666	E3 = 15	HDE3: n = 166

### 2.3. Physiochemical parameters and survival rate

Water quality criteria were precisely maintained in all tanks to ensure the well-being of the fish. A daily water exchange of 20% was performed for each tank. Water quality parameters were measured twice a day to maintain standard levels throughout the study. Aeration was provided using 120V/60Hz Airmax SilentAir LR25 pumps (USA), which delivered air through diffuser grids. In order to create microbubbles and ensure good air saturation in the water (>80%), each tank had a single rectangular diffuser grid (L × W: 1 × 0.5 ft) built with antimicrobial tubing (outer diameter: 25.4 mm, inner diameter: 12.7 mm, and airflow of 2.2 m^3^/h/meter). Every day, the tanks’ bottoms were backwashed to get rid of solid waste.

Water quality parameters, including water temperature, were closely monitored (25.39 ± 0.30–27.99 ± 0.23°C), dissolved oxygen (4.07 ± 0.31–4.98 ± 0.31 mg/L), and pH (7.34 ± 0.04–8.95 ± 0.01) were monitored twice a day by using portable meters (HI98494, Hanna, USA). Ammonia (0.89±0.08–1.64±0.22 ppm), and nitrite (0.10±0.01–0.22±0.10 mg/L) were monitored twice a week by using commercial kits (HI733, HI93708, Hanna, USA) (S1 Data). Fish in each tank were monitored twice a day for any signs of disease, abnormal behavior, or mortality. Dead fish were removed immediately if found and carefully recorded. Survival rate observed in LD, MD and HD were 100%, 100% and 98.42%, respectively due to well-maintained husbandry conditions over the study period. Mortality of only 1.58% observed in HD treatment, was due to high density. However, it was much lower than the chosen limit of 10% mortality, permitted by Animal Ethics Committee for Aquaculture trials.

### 2.4. Sample analysis

At the end of the trial, five fish were randomly sampled from each replicate across all density treatments (20 fish per treatment), resulting in a total of 180 fish being euthanized out of the 4,200 fish used in the study. The Animal Ethics Committee established a 5% population limit, which was adhered to in this sampling. For the purpose of stocking, the remaining 4,020 fish were humanely released into a neighboring lake under the management of the Department of Fisheries, Pakistan (Release Approval #: DOF/27858/2022). The fish were starved for 24 hours before to sampling. The fish were put to sleep on the day of the sampling using clove oil (0.8 ml/L of water, Sigma-Aldrich, USA), a normal dosage that puts fish to sleep in less than ten minutes.

The fish’s caudal vein was used for collecting blood into two different tubes. For hematological analysis, one tube was coated with ethylenediamine tetraacetic acid (EDTA), and the other included a clot activator for plasma collection. Following a 15-minute centrifugation of blood samples at 5,000 rpm, the plasma was separated into individual Eppendorf tubes and kept at -20°C. Each fish’s length and total body weight were noted prior to dissection. Following the fish’s dissection, gill samples were gathered, cleaned in deionized water, and kept for a full day in a 10% formaldehyde solution in preparation for histological examination. Additionally, muscle samples were obtained and kept at -20°C in accordance with the Association of Official Analytical Chemists (AOAC) [48] criteria for the examination of chemical composition, fatty acids, and amino acids. After the muscle samples were treated for additional chemical analysis, they were dried in an oven at 80°C until a constant dry weight was reached. The Kjeldahl apparatus (PCSIR Laboratories, Pakistan) was utilized to estimate crude protein, and the Soxhlet apparatus (PCSIR Laboratories, Pakistan) was employed to identify crude lipids by the Folch method [49]. A muffle furnace was used to determine the muscles’ ash content (PCSIR Laboratories, Pakistan). A fish muscle amino acid analyzer (Biochrome 30+, Biochrome Limited, Cambridge, UK) was used to measure the amount of amino acids present in the muscle [50].

Midgut intestinal samples were weighed, cleaned in deionized water, and standardized in a sterile normal saline solution containing 0.86% (1:9). After centrifuging this mixture for 15 minutes at 5,000 rpm, the supernatant was removed and kept at -20°C. For all tests, each sample was examined in duplicate. Additionally, liver tissues were obtained and homogenized for gene expression analysis at -80°C in liquid nitrogen. Condition factor (K), specific growth rate (SGR), hepatosomatic index (HSI), fish weight gain, survival rate, and feed conversion rate (FCR) were among the metrics that were estimated using certain formulas.


$$Condition\;factor\;(\%)=\frac{Total\;body\;weight\;(g)}{Total\;body\;{length\;(cm)}^{3}}\times 100$$

$$Specific\;growth\;rate\;(\%)=\frac{Ln\;final\;weight-Ln\;initial\;weight}{Time\;interval\;in\;days}\times 100$$

$$Hepatosomatic\;index=\frac{Liver\;weight}{Total\;body\;weight}\times 100$$

$$Feed\;conversion\;ratio=\frac{Weight\;of\;feed\;consumed}{Weight\;gain\;(wet\;weight)}$$

$$Survival\;rate\;(\%)=\frac{Final\;number\;of\;fish}{Initial\;stocking\;density}\times 100$$

### 2.5. Hematological analysis

Hematological parameters were measured using an auto-hematology blood analyzer (Sysmex-KX-21, Japan), calibrated specifically for fish. These parameters included hemoglobin (Hb) (g/dl), white blood cell (WBC) count (10^3^/μL) with differentiation into neutrophils (%), eosinophils (%), lymphocytes (%), and monocytes (%), red blood cell (RBC) count (10⁶/μL), and platelet count (10^3^/μL).

### 2.6. Biochemical analysis

Using a colorimetric assay kit (Thermo Fisher Scientific, USA, CAT No. EEA028), the concentration of triglycerides (TG) (mg/dl) was determined. Using an albumin kit (LOT. DR379E249; ANMOL-LAB Pvt. Ltd, India) and the bromocresol green (BCG) dye binding technique, the level of albumin (Alb) (g/dl) was measured. A commercial kit (Thermo Fisher Scientific, USA, CAT No. EEA002, E.C. 3.1.3.1) was used to measure the alkaline phosphatase (ALP) (U/L) levels. Using a commercial ELISA kit (Thermo Fisher Scientific, USA, CAT No. MAK055, E.C. 2.6.1.1), aspartate aminotransferase (AST) (U/L) was measured, and another commercial ELISA kit (Thermo Fisher Scientific, USA, CAT No. MAK052, E.C. 2.6.1.2) was used to determine alanine aminotransferase (ALT) (U/L) activity. A laboratory blood glucose analyzer (Human, Germany) was used to measure the levels of glucose (GLU) (mg/dl).

### 2.7. Cortisol assay

With a sensitivity of 1.16 ng/ml, an ELISA kit (Calbiotech, USA, CAT No. CO368S, CID 5754) was used to assess the cortisol concentration (ng/ml) in blood plasma. At 450 nm, the absorbance values were measured with a spectrophotometer.

### 2.8. Antioxidants assay

A commercial ELISA colorimetric activity kit (Thermo Fisher Scientific, USA, CAT No. EIACATC, EC 1.11.1.6) was used to measure the plasma catalase (CAT) (U/ml) activity. The activity of superoxide dismutase (SOD) was tested using an ELISA kit (PARS BIOCHME, China, CAT No. PRS-02005hu, EC 1.15.1.11), yielding results in ng/ml. A Malondialdehyde (MDA) (nmol/ml) kit (PARS BIOCHME, China, CAT No. PRS-00991hu, CAS 542-78-9) was used to measure the levels of MDA. Using an ELISA kit (PARS BIOCHME, China, CAT No. PRS-00680hu, EC 1.11.1.9), the activity of glutathione peroxidase (GPx) (IU/ml) was determined.

### 2.9. Digestive enzymes assay

The supernatant of processed midgut intestinal samples was utilized for studies of digestive enzymes. Using a commercial ELISA kit (Sigma Aldrich, USA, CAT No. MAK046, EC 3.1.1.3), the activity of lipase (U/L) was determined. Another commercial ELISA kit (Sigma Aldrich, USA, CAT No. MAK009A, EC 3.2.1.1) was used to measure the amylase (U/L) activity. Protease activity was measured using 1% casein as the substrate in 0.2 M phosphate buffer at pH 7.0, in accordance with the procedure outlined in reference [51]. The amount of enzyme that releases 1 μg/ml/min of tyrosine, measured at 660 nm, is known as one unit of protease activity.

### 2.10. Histological analysis

The gills were fixed in 10% buffered formalin for a minimum of 24 hours to prevent cellular autolysis. Preserved gill samples were dehydrated using a series of alcohol gradient (70%, 90% and 100%) and xylene. For wax embedding, gills were processed in paraffin wax. Sections were cut using a microtome (Bio-Equip, China), with wax blocks trimmed to 10 μm and transverse sections cut to 4 μm thickness. Dewaxing was performed with xylene and alcohol, followed by staining with hematoxylin and eosin. The stained gill sections were mounted with DPX (a mixture of distyrene, plasticizer, and xylene) (Merck, Germany) [52]. Microphotographs were taken using a digital camera fitted to an optical microscope (Trinocular E-200, Nikon, Japan). Histological analysis of gills was performed to determine the alteration in gills structure like

- Alteration in filament
- Alteration in lamella structure
- Degeneration of primary lamella
- Degeneration of secondary lamella
- Epithelial lifting
- Edema
- Tissue debris
- Lamellar fusion
- Edema
- Hyperplasia.

### 2.11. Gene expression analysis

Total RNA was extracted from 50 mg of liver tissues at 37°C using the Trizol technique (Catalog No. 15596026, Thermo, USA). A Thermo Nanodrop 2000 spectrophotometer was used to confirm the quantity and purity of the RNA (Waltham, MA, USA). The SuperScript III First-Strand cDNA Synthesis Kit (Cat. No. 18080051, Life Technologies) was used to synthesise first strand cDNA. There were 5.0 μg of total RNA used for cDNA synthesis. A 20 μl total volume of poly-A tail primed oligo(dT) was used for the synthesis. The following is how the initial reaction mixture was made:

- 5 μg RNA
- 1 μL 50 μM oligo(dT)20
- 1 μL 10 mM dNTP mix
- Water to a total volume of 10 μL

For five minutes, this mixture was incubated at 65°C. To create the cDNA synthesis Mix-2, add the following:

- 2 μL 10X RT buffer
- 4 μL 25 mM MgCl₂
- 2 μL 0.1 M DTT
- 1 μL RNaseOUT™ (40 U/μL)
- 1 μL SuperScript® III RT (200 U/μL)
- Total volume of 10 μL

Each RNA/primer mixture received 10 μL of the cDNA synthesis mixture, which was then carefully mixed in and collected using a quick centrifugation. After that, the tube was incubated for 50 minutes at 50°C. After that, the process was stopped by heating it for five minutes at 85°C. At -20°C, the resultant cDNA was kept. Using the Primer Quest program from Integrated DNA Technologies, 2 μL of the cDNA template was utilized in a separate tube together with gene-specific primers (forward and reverse) for the PCR reaction Table 3. A concentration of 1 μL (10 μM) of each primer was utilized, in addition to 12.5 μL of the Maxi SYBR Green/ROX qPCR Master Mix (2X) SYBR Green PCR Master Mix.

**Table 3:** Primer sequence of genes.

#	Gene	Accession no.	Primer sequence
01	Somatostatin-1 (SST-1) F	XP_005464163.1	TGCTGGGCTCCAAACAG
02	Somatostatin-1 (SST-1) R	XP_005464163.1	AGGGAAGTTCTCCTCTTCCA
03	Interleukin 1-β (IL-1β) F	XP_019221389.1	TGGAGGAGGTGACGGATAAA
04	Interleukin 1-β (IL-1β) R	XP_019221389.1	CAGTGTCGCGTTTGTAGAAGA
05	Proopiomelanocortin (POMC-α) F	XP_003457991.1	CTCCTACTCAATGGAGCACTTC
06	Proopiomelanocortin (POMC-α) R	XP_003457991.1	AAGCTCTCGTCTCCTCATCT
07	β-Actin-F	ABK20357.1	GAGGTATCCTGACCCTGAAGTA
08	β -Actin-R	ABK20357.1	ACTCTCAGCTCGTTGTAGGA

*F: Forward primer; R: Reverse primer

The PCR conditions were as follows:

- Initial denaturation: 95°C for 2 minutes
- Denaturation: 95°C for 15 seconds
- Annealing: 55°C for 1 minute
- Extension: 72°C for 1 minute

The housekeeping gene for reference was β-Actin. Relative quantification utilizing the ΔΔCT method allowed for the determination of the 2-fold induction.

### 2.12. Statistical analysis

Statistical analyses were performed using SPSS v.29 software, with data presented as Mean ± SE. The Levene test was used to assess homogeneity of variance. Two-Way ANOVA determined the effects of stocking density and calcium disodium ethylene diamine tetraacetic acid (EDTA) supplementation dose on various parameters, with a significance level set at 0.05. Degrees of freedom (df) were calculated as follows: for stocking density, df was 2 (three treatments); for EDTA supplementation, df was 3 (four doses per density treatment); and for the interaction between EDTA and density, df was 10 (2 × 5).

## 3. Results

### 3.1. Growth

A significant difference (P<0.05) was observed in total body length (df_2_, F = 30.67), total body weight (df_2_, F = 45.29), condition factor (df_2_, F = 23.62), specific growth rate (df_2_, F = 47.22), hepatosomatic index (df_2_, F = 51.43) and viscerosomatic index (df_2_, F = 23.32) between three density treatments (LDE, MDE, HDE) Table 4. A significant variation (P<0.05) in total body length (df_3_, F = 2.73), total body weight (df_3_, F = 9.31) condition factor (df_3,_ F = 4.67), specific growth rate (df_3,_ F = 8.88), viscerosomatic index (df_3,_ F = 6.81) and hepatosomatic index (df_3,_ F = 5.52) were observed across different levels of EDTA supplementation (four in each treatment) within each treatment. Other than this, the interactive effect of two independent factors i.e., stocking density and EDTA concentration (density*EDTA concentration) was also observed. The combined effect of density*EDTA concentration on total body length (df_6_, F = 2.24), total body weight (df_6_, F = 3.17), condition factor (df_6_, F = 2.75), specific growth rate (df_6_, F = 3.08) and hepatosomatic index (df_6_, F = 23.30) was noted to be significant (P<0.05) between three density treatments. However, in case of viscerosomatic index, the combined effect of density*EDTA concentration between three density treatments was found to be insignificant (df_6_, F = 1.39, P>0.05). The survival rate of fish in both LSD and MSD treatments was 100.00% but in HSD treatment, it was within the range of 98.00% - 99.30%.

**Table 4:** Three density treatments (LDE, MDE, HDE) were analyzed (Mean ± SE) for total body length (cm), total body weight (g), specific growth rate (%), condition factor (%), hepatosomatic index (%), viscerosomatic index (%), FCR, and survival rate (%), with each treatment having four levels of EDTA supplementation (E0 = 0 g/kg, E1 = 5 g/kg, E2 = 10 g/kg, E3 = 15 g/kg).

Treatment	TBL (cm)	TBW (g)	SGR (%)	K (%)	HSI (%)	VSI (%)	FCR	Survival rate (%)
**LDE0**	8.55±0.25^**B**,a^	89.50±1.12 ^**B**,bc^	1.82±1.20 ^**B**,bc^	3.35±0.26 ^**A**,b^	0.79±0.03 ^**A**,bc^	3.45±1.11 ^**A**,a^	0.61±0.36 ^**A**,a^	100.00±0.01 ^**A**,a^
**LDE1**	9.05±0.26 ^**B**,a^	90.50±0.92 ^**B**,c^	1.84±1.40 ^**B**,c^	3.46±0.55 ^**A**,a^	0.89±0.04 ^**A**,a^	3.48±1.21 ^**A**,ab^	0.60±0.45 ^**A**,a^	100.00±0.01 ^**A**,a^
**LDE2**	9.08±0.37 ^**B**,a^	85.00±1.23 ^**B**,ab^	1.74±1.60 ^**B**,ab^	3.97±0.66 ^**A**,ab^	0.87±0.05 ^**A**,ab^	3.92±1.01 ^**A**,b^	0.65±0.43 ^**A**,a^	100.00±0.02 ^**A**,a^
**LDE3**	8.53±0.47 ^**B**,a^	84.75±1.14 ^**B**,a^	1.73±1.60 ^**B**,a^	3.85±0.80 ^**A**,ab^	0.96±0.03 ^**A**,c^	3.65±.102 ^**A**,b^	0.66±0.45 ^**A**,a^	100.00±0.03 ^**A**,a^
**MDE0**	8.83±0.38 ^**B**,a^	90.00±1.24 ^**B**,bc^	1.83±1.40 ^**B**,bc^	3.33±0.69 ^**A**,b^	1.19±0.02 ^**B**,bc^	3.43±1.08 ^**A**,a^	0.60±0.55 ^**A**,a^	100.00±0.04 ^**A**,a^
**MDE1**	8.60±0.25 ^**B**,a^	88.00±1.35 ^**B**,c^	1.79±1.50 ^**B**,c^	3.22±0.50 ^**A**,a^	0.90±0.04 ^**B**,a^	3.64±0.97 ^**A**,ab^	0.62±0.64 ^**A**,a^	100.00±0.02 ^**A**,a^
**MDE2**	8.50±0.25 ^**B**,a^	84.00±1.73 ^**B**,ab^	1.72±1.60 ^**B**,ab^	3.87±0.30 ^**A**,ab^	0.94±0.03 ^**B**,ab^	3.62±0.99 ^**A**,b^	0.67±0.42 ^**A**,a^	100.00±0.03 ^**A**,a^
**MDE3**	8.63±0.25 ^**B**,a^	83.75±1.83 ^**B**,a^	1.71±1.77 ^**B**,a^	3.16±0.50 ^**A**,ab^	0.98±0.03 ^**B**,c^	3.92±1.34 ^**A**,b^	0.67±0.24 ^**A**,a^	100.00±0.05 ^**A**,a^
**HDE0**	7.60±0.33 ^**A**,a^	77.00±1.23 ^**A**,bc^	1.57±1.67 ^**A**,bc^	3.72±0.50 ^**B**,b^	0.97±0.05 ^**B**,bc^	3.84±1.24 ^**B**,a^	0.77±0.36 ^**B**,b^	98.00±0.06 ^**B**,b^
**HDE1**	8.38±0.23^**A**,a^	83.00±1.64 ^**A**,c^	1.70±1.54 ^**A**,c^	3.21±0.67 ^**B**,a^	1.02±0.04 ^**B**,a^	4.12±1.30 ^**B**,ab^	0.68±0.44 ^**B**,b^	99.30±0.08 ^**B**,b^
**HDE2**	7.78±0.35^**A**,a^	79.75±1.64 ^**A**,ab^	1.63±1.54 ^**A**,ab^	3.43±0.77 ^**B**,ab^	1.02±0.03 ^**B**,ab^	4.24±1.43 ^**B**,b^	0.72±0.43 ^**B**,b^	98.30±0.09 ^**B**,b^
**HDE3**	7.78±0.35^**A**,a^	79.00±1.54 ^**A**,a^	1.61±1.57 ^**A**,a^	3.25±0.45 ^**B**,ab^	1.03±0.05 ^**B**,c^	4.20±1.13 ^**B**,b^	0.73±0.36 ^**B**,b^	98.10±0.05 ^**B**,b^

Significant differences (P < 0.05) among the three density treatments (LDE, MDE, HDE) are marked by bold, uppercase red superscripts, while differences between the four EDTA supplementation levels within each density treatment are indicated by lowercase superscripts.

### 3.2. Chemical composition of muscles

The content of moisture (df_2_, F = 1547.58), crude protein (df_2_, F = 3675.00), crude ash (df_2_, F = 1456.00) and crude fat (df_2_, F = 2227.75) was significantly different (P<0.05) between three density treatments Table 5. A significant difference (P<0.05) in the content of moisture (df_3_, F = 1866.66), crude protein (df_3_, F = 855.55), crude ash (df_3_, F = 1576.55) and crude fat (df_3_, F = 3816.36) was observed between different levels of EDTA supplementation (four in each treatment) in each density treatment. The interactive effect of density*EDTA concentration on moisture (df_6_, F = 730.91), crude protein (df_6_, F = 2430.55), crude ash (df_6_, F = 1051.55) and crude fat (df_6_, F = 3518.86) between three density treatments was also noted to be significant (P<0.05).

**Table 5:** The proximate composition (%) of muscle samples was analyzed (mean ± SE) for each of the three density treatments (LDE, MDE, and HDE), each of which had four levels of EDTA supplementation (E0 = 0 g/kg, E1 = 5 g/kg, E2 = 10 g/kg, and E3 = 15 g/kg).

Trt	Moisture (%)	Crude protein (%)	Crude fat (%)	Crude ash (%)
**LDE0**	74.10±0.33 ^**B**,b^	18.60±0.32 ^**B**,a^	1.90±0.43 ^**B**,c^	5.50±0.60 ^**A**,c^
**LDE1**	74.12±0.35 ^**B**,c^	18.48±0.23 ^**B**,c^	1.91±0.54 ^**B**,b^	5.69±0.65 ^**A**,b^
**LDE2**	73.90±0.54 ^**B**,a^	18.70±0.32 ^**B**,c^	1.87±0.65 ^**B**,d^	5.54±0.54 ^**A**,a^
**LDE3**	74.30±0.35 ^**B**,c^	18.90±0.42 ^**B**,b^	1.20±0.45 ^**B**,a^	5.60±0.45 ^**A**,d^
**MDE0**	74.11±0.23 ^**B**,b^	18.39±0.34 ^**A**,a^	1.70±0.54 ^**C**,c^	5.80±0.55 ^**B**,c^
**MDE1**	74.20±0.76 ^**B**,c^	18.65±0.23 ^**A**,c^	1.65±0.34 ^**C**,b^	5.49±0.46 ^**B**,b^
**MDE2**	74.03±0.76 ^**B**,a^	18.50±0.34 ^**A**,c^	1.56±0.45 ^**C**,d^	5.51±0.44 ^**B**,a^
**MDE3**	74.00±0.60 ^**B**,c^	18.52±0.54 ^**A**,b^	1.78±0.43 ^**C**,a^	5.70±0.56 ^**B**,d^
**HDE0**	73.99±0.65 ^**A**,b^	18.71±0.43 ^**C**,a^	1.58±0.76 ^**A**,c^	5.81±0.65 ^**C**,c^
**HDE1**	74.02±0.65 ^**A**,c^	18.92±0.33 ^**C**,c^	1.48±0.34 ^**A**,b^	5.58±0.35 ^**C**,b^
**HDE2**	73.80±0.55 ^**A**,a^	18.90±0.44 ^**C**,c^	1.69±0.65 ^**A**,d^	5.61±0.75 ^**C**,a^
**HDE3**	74.05±0.50 ^**A**,c^	18.48±0.43 ^**C**,b^	1.62±0.44 ^**A**,a^	5.85±0.76 ^**C**,d^

* Trt: Treatments; Significant differences (P < 0.05) among the three density treatments (LDE, MDE, HDE) are marked by bold, uppercase red superscripts, while differences between the four EDTA supplementation levels within each density treatment are indicated by lowercase superscripts.

### 3.3. Profile of amino acids

A significant difference (P<0.05) was observed in the content of methionine (df_2_, F = 48260.33), threonine (df_2_, F = 74393.08), valine (df_2_, F = 17791.08), isoleucine (df_2_, F = 3498.25), leucine (df_2_, F = 65941.75), phenylalanine (df_2_, F = 48294.75) and histidine (df_2_, F = 14092.75) between three density treatments Table 6. Similarly, the content of lysine (df_2_, F = 101284.75), arginine (df_2_, F = 30641.33), ornithine (df_2_, F = 2668.75), cysteine (df_2_, F = 3417.75), aspartic acid (df_2_, F = 321811.00), asparagine (df_2_, F = 136598.58) and serine (df_2_, F = 319508.58) also differed significantly (P<0.05) between three density treatments. A significant difference (P<0.05) was observed in the content of glutamic acid (df_2_, F = 402970.75), glycine (df_2_, F = 46239.08), alanine (df_2_, F = 42064.75), proline (df_2_, F = 13073.08), glutamine (df_2_, F = 400569.75) and tyrosine (df_2_, F = 24199.00) between three density treatments (LDE, MDE, HDE).

**Table 6:** Muscle samples from three density treatments (LDE, MDE, HDE) were analyzed (Mean ± SE) for amino acids (%), with each treatment having four EDTA supplementation levels (E0 = 0 g/kg, E1 = 5 g/kg, E2 = 10 g/kg, and E3 = 15 g/kg). The results are given as milligrams of amino acid (mg/gcp) per gram of crude protein.

Essential amino acids										
Trt	Methionine	Threonine	Valine	Isoleucine	Leucine	Phenylalanine	Histidine	Lysine	Arginine	Ornithine
**LDE0**	21.48±0.43 ^**A**,a^	36.57±0.55 ^**A**,a^	37.13±0.56 ^**C**,d^	34.20±0.65 ^**C**,a^	60.59±0.64 ^**B**,a^	33.58±0.54 ^**C**,b^	22.70±0.65 ^**C**,b^	56.48±0.54 ^**C**,b^	49.71±0.54 ^**A**,b^	2.36±0.54 ^**A**,c^
**LDE1**	22.82±0.43 ^**A**,c^	38.60±0.32 ^**A**,d^	37.14±0.65 ^**C**,a^	34.70±0.44 ^**C**,c^	61.61±0.53 ^**B**,d^	33.37±0.54 ^**C**,d^	22.28±0.45 ^**C**,c^	59.37±0.56 ^**C**,c^	49.62±0.56 ^**A**,d^	2.25±0.43^**A**,b^
**LDE2**	22.60±0.46 ^**A**,b^	37.17±0.43 ^**A**,b^	37.38±0.44 ^**C**,c^	33.62±0.33 ^**C**,b^	59.62±0.64 ^**B**,c^	32.58±0.34 ^**C**,a^	21.83±0.43 ^**C**,a^	57.69±0.44 ^**C**,a^	48.58±0.76 ^**A**,a^	2.55±0.65 ^**A**,b^
**LDE3**	23.60±0.66 ^**A**,d^	36.59±0.23 ^**A**,c^	36.38±0.54 ^**C**,b^	35.49±0.22 ^**C**,d^	58.70±0.51 ^**B**,b^	34.59±0.34 ^**C**,c^	22.82±0.32 ^**C**,d^	56.68±0.45 ^**C**,d^	51.61±0.55 ^**A**,c^	2.15±0.43 ^**A**,a^
**MDE0**	23.25±0.45 ^**B**,a^	38.05±0.12 ^**C**,a^	36.58±0.65 ^**B**,d^	32.46±0.55 ^**A**,a^	58.72±0.48 ^**C**,a^	31.78±0.23 ^**A**,b^	20.69±0.34 ^**A**,b^	55.25±0.45 ^**B**,b^	49.26±0.45 ^**B**,b^	2.71±0.34 ^**C**,c^
**MDE1**	24.48±0.66 ^**B**,c^	39.24±0.45 ^**C**,d^	35.71±0.75 ^**B**,a^	35.77±0.60 ^**A**,c^	61.57±0.66 ^**C**,d^	32.38±0.34 ^**A**,d^	22.71±0.54 ^**A**,c^	55.84±0.34 ^**B**,c^	52.93±0.34 ^**B**,d^	2.82±0.46 ^**C**,b^
**MDE2**	21.60±0.54 ^**B**,b^	37.05±0.65 ^**C**,b^	37.26±0.65 ^**B**,c^	34.26±0.65 ^**A**,b^	60.83±0.76 ^**C**,c^	33.48±0.23 ^**A**,a^	22.28±0.34 ^**A**,a^	56.28±0.45 ^**B**,a^	49.16±0.23 ^**B**,a^	2.28±0.64 ^**C**,b^
**MDE3**	22.91±0.55 ^**B**,d^	38.49±0.55 ^**C**,c^	37.06±0.54 ^**B**,b^	34.63±0.62 ^**A**,d^	61.91±0.74 ^**C**,b^	33.34±0.34 ^**A**,c^	22.20±0.43 ^**A**,d^	59.20±0.65 ^**B**,d^	48.73±0.56 ^**B**,c^	2.20±0.54 ^**C**,a^
**HDE0**	22.69±0.76 ^**C**,a^	37.25±0.66 ^**B**,a^	37.49±0.65 ^**A**,d^	33.70±0.65 ^**B**,a^	59.81±0.64 ^**A**,a^	32.80±0.34 ^**B**,b^	21.91±0.45 ^**B**,b^	57.87±0.34 ^**A**,b^	48.45±0.56 ^**C**,b^	2.57±0.34 ^**B**,c^
**HDE1**	23.38±0.44 ^**C**,c^	36.81±0.76 ^**B**,d^	36.26±0.54 ^**A**,a^	35.26±0.75 ^**B**,c^	58.84±0.64 ^**A**,d^	34.62±0.23 ^**B**,d^	22.73±0.65 ^**B**,c^	56.39±0.35 ^**A**,c^	51.28±0.23 ^**C**,d^	2.28±0.42 ^**B**,b^
**HDE2**	23.38±0.55 ^**C**,b^	38.81±0.55 ^**B**,b^	36.50±0.54 ^**A**,c^	32.82±0.63 ^**B**,b^	59.03±0.74 ^**A**,c^	31.95±0.54 ^**B**,a^	20.93±0.45 ^**B**,a^	55.47±0.35 ^**A**,a^	49.37±0.45 ^**C**,a^	2.50±0.44 ^**B**,b^
**HDE3**	24.37±0.45 ^**C**,d^	39.17±0.76 ^**B**,c^	35.83±0.65 ^**A**,b^	35.71±0.54 ^**B**,d^	61.53±0.54 ^**A**,b^	32.27±0.45 ^**B**,c^	22.84±0.76 ^**B**,d^	55.95±0.56 ^**A**,d^	52.94±0.65 ^**C**,c^	2.61±0.42 ^**B**,a^
**Non-Essential amino acids**										
**Trt**	**Cysteine**	**Aspartic acid**	**Asparagine**	**Serine**	**Glutamic acid**	**Glycine**	**Alanine**	**Proline**	**Tyrosine**	**Glutamine**
**LDE0**	8.73±0.43 ^**C**,a^	63.81±0.43 ^**A**,c^	56.13±0.43 ^**A**,b^	36.55±0.54 ^**C**,d^	112.57±0.62 ^**B**,a^	55.23±0.55 ^**C**,d^	58.80±0.72 ^**A**,d^	39.15±0.54 ^**A**,b^	20.35±0.26 ^**A**,c^	101.57±1.11 ^**B**,a^
**LDE1**	8.57±0.23 ^**C**,d^	63.61±0.32 ^**A**,a^	55.83±0.32 ^**A**,c^	35.73±0.46 ^**C**,b^	109.89±0.45 ^**B**,d^	52.87±0.65 ^**C**,b^	55.86±0.70 ^**A**,a^	39.77±0.54 ^**A**,d^	18.84±0.23 ^**A**,a^	98.91±1.23 ^**B**,d^
**LDE2**	8.65±0.12 ^**C**,c^	63.66±0.34 ^**A**,d^	55.29±0.34 ^**A**,a^	36.50±0.46 ^**C**,c^	111.82±0.54 ^**B**,b^	54.76±0.73 ^**C**,c^	58.65±0.51 ^**A**,c^	39.49±0.65 ^**A**,a^	20.61±0.23 ^**A**,b^	100.82±1.64 ^**B**,b^
**LDE3**	8.13±0.21 ^**C**,b^	60.93±0.45 ^**A**,b^	60.49±0.34 ^**A**,d^	31.85±0.45 ^**C**,a^	118.75±0.61 ^**B**,c^	52.87±0.61 ^**C**,a^	54.91±0.52 ^**A**,b^	39.73±0.64 ^**A**,c^	19.87±0.24 ^**A**,d^	107.75±1.68 ^**B**,c^
**MDE0**	8.12±0.22 ^**B**,a^	65.39±0.54 ^**C**,c^	54.50±0.56 ^**B**,b^	30.53±0.83 ^**B**,d^	108.63±0.34 ^**A**,a^	51.65±0.61 ^**B**,d^	55.67±0.41 ^**C**,d^	38.78±0.76 ^**B**,b^	19.33±0.25 ^**B**,c^	97.62±1.56 ^**A**,a^
**MDE1**	8.51±0.32 ^**B**,d^	67.47±0.44 ^**C**,a^	61.47±0.45 ^**B**,c^	33.48±0.64 ^**B**,b^	114.60±0.43 ^**A**,d^	53.52±0.52 ^**B**,b^	60.70±0.55 ^**C**,a^	41.90±0.65 ^**B**,d^	21.42±0.24 ^**B**,a^	103.60±1.56 ^**A**,d^
**MDE2**	8.25±0.12 ^**B**,c^	63.63±0.45 ^**C**,d^	56.06±0.54 ^**B**,a^	34.63±0.54 ^**B**,c^	112.49±0.64 ^**A**,b^	55.20±0.48 ^**B**,c^	58.92±0.66 ^**C**,c^	39.06±0.55 ^**B**,a^	20.35±0.29 ^**B**,b^	101.50±1.67 ^Ab^
**MDE3**	8.45±0.23 ^**B**,b^	64.02±0.54 ^**C**,b^	55.94±0.34 ^**B**,d^	35.51±0.43 ^**B**,a^	109.80±0.63 ^**A**,c^	52.94±0.51 ^**B**,a^	56.03±0.77 ^**C**,b^	39.75±0.76 ^**B**,c^	18.95±0.26 ^**B**,d^	98.81±1.34 ^**A**,c^
**HDE0**	8.03±0.12 ^**A**,a^	63.44±0.55 ^**B**,c^	55.57±0.23 ^**C**,b^	36.68±0.64 ^**A**,d^	111.75±0.63 ^**C**,a^	54.73±0.65 ^**A**,d^	58.71±0.87 ^**B**,d^	39.48±0.70 ^**C**,b^	20.71±0.24 ^**C**,c^	100.75±1.43 ^**C**,a^
**HDE1**	8.35±0.32 ^**A**,d^	60.73±0.45 ^**B**,a^	60.93±0.32 ^**C**,c^	31.94±0.46 ^**A**,b^	118.95±0.44 ^**C**,d^	52.95±0.63 ^**A**,b^	55.04±0.89 ^**B**,a^	39.83±0.65 ^**C**,d^	19.93±0.23 ^**C**,a^	107.95±1.34 ^**C**,d^
**HDE2**	8.35±0.34 ^**A**,c^	65.71±0.34 ^**B**,d^	54.32±0.45 ^**C**,a^	30.85±0.43 ^**A**,c^	108.95±0.55 ^**C**,b^	51.50±0.81 ^**A**,c^	55.39±0.88 ^**B**,c^	38.50±0.44 ^**C**,a^	19.29±0.22 ^**C**,b^	97.95±1.32 ^**C**,b^
**HDE3**	8.57±0.23 ^**A**,b^	67.37±0.65 ^**B**,b^	61.87±0.34 ^**C**,d^	33.08±0.41 ^**A**,a^	114.80±0.62 ^**C**,c^	53.46±0.51 ^**A**,a^	60.69±0.55 ^**B**,b^	41.94±0.50 ^**C**,c^	21.94±0.33 ^**C**,d^	103.81±1.43 ^**C**,c^

* Trt: Treatments; Significant differences (P < 0.05) among the three density treatments (LDE, MDE, HDE) are marked by bold, uppercase red superscripts, while differences between the four EDTA supplementation levels within each density treatment are indicated by lowercase superscripts.

The effect of different levels of EDTA supplementation on methionine (df_3_, F = 83931.55), threonine (df_3_, F = 36966.22), valine (df_3_, F = 30605.55), isoleucine (df_3_, F = 214863.44), leucine (df_3_, F = 60669.77), phenylalanine (df_3_, F = 37662.13), histidine (df_3_, F = 53728.88), lysine (df_3_, F = 37778.80), arginine (df_3_, F = 309270.88) was found to be significant (P<0.05) within each density treatment. Similarly, in case of ornithine (df_3_, F = 1812.22), cysteine (df_3_, F = 1231.02), aspartic acid (df_3_, F = 5949.02), asparagine (df_3_, F = 1183558.44), serine (df_3_, F = 47876.88), glutamic acid (df_3_, F = 823948.22), glycine (df_3_, F = 38784.66), alanine (df_3_, F = 16657.47) and proline (df_3_, F = 139695.88), the effect of different levels of EDTA supplementation within each density treatment was noted to be significant (P<0.05). The content of glutamine (df_3_, F = 821369.91) and tyrosine (df_3_, F = 1526.58) also differed significantly (P<0.05) across different levels of EDTA supplementation within each density treatment.

The combined effect of density*EDTA concentration on methionine (df_6_, F = 50674.55), threonine (df_6_, F = 89309.30), valine (df_6_, F = 25282.63), isoleucine (df_6_, F = 43989.36), leucine (df_6_, F = 148859.86), phenylalanine (df_6_, F = 77551.63) histidine (df_6_, F = 34851.63), lysine (df_6_, F = 187794.63), arginine (df_6_, F = 175178.88) and ornithine (df_6_, F = 4203.30) was found to be significant (P<0.05) between three density treatments. The interactive effect of density*EDTA concentration on cysteine (df_6_, F = 4764.86), aspartic acid (df_6_, F = 459851.77), asparagine (df_6_, F = 438866.36), serine (df_6_, F = 540136.13), glutamic acid (df_6_, F = 997510.30), glycine (df_6_, F = 172969.41), alanine (df_6_, F = 587667.30), proline (df_6_, F = 76221.63), glutamine (df_3_, F = 989335.26) and tyrosine (df_3_, F = 109160.33) was found to be significant (P<0.05) between three density treatments. The content of glutamine (df_3_, F = 821369.91) and tyrosine (df_3_, F = 1526.58) also differed significantly (P<0.05) across different levels of EDTA supplementation within each density treatment.

### 3.4. Profile of fatty acids

#### 3.4.1. Saturated fatty acids

A significant difference (P<0.05) was observed in myristic acid (df_2_, F = 16653.00), pentadecylic acid (df_2_, F = 121.33), palmitic acid (df_2_, F = 32940.25), margaric acid (df_2_, F = 529.08), stearic acid (df_2_, F = 6974.33) between three density treatments Table 7. The effect of varying levels of EDTA (E) supplementation on myristic acid (df_3_, F = 5563.25), pentadecylic acid (df_3,_ F = 19.25), palmitic acid (df_3,_ F = 38281.44), margaric acid (df_3,_ F = 101.88) and stearic acid (df_3,_ F = 24021.66) was noted to be significant (P<0.05) within each density treatment. The combined effect of density*EDTA concentration on myristic acid (df_6_, F = 12432.00), pentadecylic acid (df_6,_ F = 413.00), palmitic acid (df_6,_ F = 24823.36), margaric acid (df_6,_ F = 101.30), stearic acid (df_6,_ F = 14725.66) was found to be significant (P<0.05) between three density treatments.

**Table 7:** Four EDTA supplementation levels (E0 = 0 g/kg, E1 = 5 g/kg, E2 = 10 g/kg, and E3 = 15 g/kg) were applied to each of the three density treatments (LDE, MDE, and HDE) for which the analysis of fatty acids in total lipids extracted from muscle samples was done (Mean ± SE). The values are given as a percentage (%) of the total fats in the body.

**Saturated fatty acids (SFA)**						
**Trt**	**Myristic acid**	**Pentadecylic acid**	**Palmitic acid**	**Margaric acid**	**Stearic acid**	
**LDE0**	3.15±0.21 ^**A**,d^	1.33±0.03 ^**B**,b^	32.46±0.33 ^**C**,b^	0.43±0.02 ^**B**,c^	6.43±0.11 ^**C**,b^	
**LDE1**	3.02±0.12 ^**A**,b^	1.20±0.03 ^**B**,c^	33.23±0.44 ^**C**,c^	0.40±0.03 ^**B**,b^	6.76±0.12 ^**C**,d^	
**LDE2**	3.25±0.11 ^**A**,c^	1.23±0.04 ^**B**,a^	33.33±0.55 ^**C**,a^	0.35±0.01 ^**B**,c^	6.42±0.13 ^**C**,a^	
**LDE3**	2.51±0.12 ^**A**,a^	1.38±0.04 ^**B**,c^	34.43±0.60 ^**C**,d^	0.33±0.09 ^**B**,a^	7.56±0.14 ^**C**,c^	
**MDE0**	3.85±0.13 ^**B**,d^	1.32±0.05 ^**C**,b^	32.43±0.45 ^**B**,b^	0.42±0.03 ^**C**,c^	6.24±0.16^**A**,b^	
**MDE1**	3.48±0.30 ^**B**,b^	1.33±0.05 ^**C**,c^	33.43±0.54 ^**B**,c^	0.37±0.04 ^**C**,b^	6.55±0.13 ^**A**,d^	
**MDE2**	3.03±0.12 ^**B**,c^	1.31±0.04 ^**C**,a^	32.19±0.30 ^**B**,a^	0.45±0.05 ^**C**,c^	6.37±0.15 ^**A**,a^	
**MDE3**	3.01±0.23 ^**B**,a^	1.22±0.06 ^**C**,c^	33.13±0.43 ^**B**,d^	0.35±0.03 ^**C**,a^	6.81±0.17 ^**A**,c^	
**HDE0**	3.37±0.32 ^**C**,d^	1.20±0.06 ^**A**,b^	33.23±0.42 ^**A**,b^	0.31±0.03 ^**A**,c^	6.35±0.16 ^**B**,b^	
**HDE1**	2.90±0.32 ^**C**,b^	1.32±0.09 ^**A**,c^	32.27±0.47 ^**A**,c^	0.28±0.02 ^**A**,b^	7.51±0.16 ^**B**,d^	
**HDE2**	3.70±0.12 ^**C**,c^	1.25±0.08 ^**A**,a^	32.35±0.32 ^**A**,a^	0.35±0.02 ^**A**,c^	6.14±0.16 ^**B**,a^	
**HDE3**	3.81±0.23 ^**C**,a^	1.25±0.07 ^**A**,c^	33.13±0.40 ^**A**,d^	0.32±0.03 ^**A**,a^	6.23±0.19 ^**B**,c^	
**Monounsaturated fatty acids (MUFA)**						
**Trt**	**Tetrasenoic acid**	**Pentadecenoic acid**	**Palmitoleic acid**	**Heptadecenoic acid**	**Oleic acid**	
**LDE0**	0.41±0.02 ^**C**,a^	0.55±0.09 ^**C**,c^	11.75±0.13 ^**B**,c^	1.52±0.09 ^**B**,b^	20.20±0.22 ^**B**,b^	
**LDE1**	0.42±0.03 ^**C**,d^	0.48±0.04 ^**C**,a^	12.54±0.15 ^**B**,a^	1.63±0.08 ^**B**,c^	19.80±0.23 ^**B**,c^	
**LDE2**	0.34±0.04 ^**C**,b^	0.63±0.03 ^**C**,b^	10.77±0.16 ^**B**,d^	1.61±0.06 ^**B**,a^	17.77±0.24 ^**B**,a^	
**LDE3**	0.68±0.05 ^**C**,c^	0.56±0.22 ^**C**,c^	9.83±0.11 ^**B**,b^	1.65±0.09 ^**B**,d^	20.48±0.20 ^**B**,d^	
**MDE0**	0.43±0.05 ^**B**,a^	0.57±0.23 ^**B**,c^	10.79±0.11 ^**C**,c^	1.62±0.07 ^**A**,b^	19.58±0.22 ^**C**,b^	
**MDE1**	0.47±0.06 ^**B**,d^	0.60±0.24 ^**B**,a^	10.54±0.14 ^**C**,a^	1.60±0.05 ^**A**,c^	19.79±0.22 ^**C**,c^	
**MDE2**	0.46±0.08 ^**B**,b^	0.45±0.24 ^**B**,b^	11.65±0.15 ^**C**,d^	1.42±0.05 ^**A**,a^	20.44±0.24 ^**C**,a^	
**MDE3**	0.35±0.08 ^**B**,c^	0.58±0.13 ^**B**,c^	12.46±0.16 ^**C**,b^	1.60±0.04 ^**A**,d^	19.90±0.23 ^**C**,d^	
**HDE0**	0.30±0.06 ^**A**,a^	0.60±0.32 ^**A**,c^	10.67±0.17 ^**A**,c^	1.58±0.03 ^**A**,b^	17.87±0.26 ^**A**,b^	
**HDE1**	0.58±0.06 ^**A**,d^	0.46±0.21 ^**A**,a^	9.14±0.18 ^**A**,a^	1.54±0.03 ^**A**,c^	20.38±0.24 ^**A**,c^	
**HDE2**	0.37±0.04 ^**A**,b^	0.53±0.22 ^**A**,b^	10.89±0.21 ^**A**,d^	1.56±0.06 ^**A**,a^	19.38±0.32 ^**A**,a^	
**HDE3**	0.33±0.04 ^**A**,c^	0.52±0.32 ^**A**,c^	10.34±0.20 ^**A**,b^	1.55±0.04 ^**A**,d^	19.89±0.43 ^**A**,d^	
**Polyunsaturated fatty acids (PUFA)**						
**Trt**	**Linoleic acid**	**Eicosadienoic acid**	**α-linolenic acid**	**Eicosapentanoic acid**	**Decosapentanoic acid**	**Decosahexanoic acid**
**LDE0**	5.83±0.22 ^**C**,b^	2.81±0.11 ^**C**,c^	12.71±0.19 ^**C**,a^	6.54±0.11 ^**B**,b^	7.63±0.11 ^**B**,a^	4.88±0.16 ^**A**,b^
**LDE1**	6.35±0.11 ^**C**,c^	2.78±0.13 ^**C**,b^	12.78±0.13 ^**C**,c^	6.19±0.12 ^**B**,c^	7.32±0.13 ^**B**,d^	4.46±0.14 ^**A**,d^
**LDE2**	6.49±0.09 ^**C**,d^	2.64±0.12 ^**C**,d^	8.64±0.14 ^**C**,d^	4.70±0.14 ^**B**,a^	5.66±0.14 ^**B**,b^	4.47±0.15 ^**A**,a^
**LDE3**	5.82±0.15 ^**C**,a^	2.59±0.12 ^**C**,a^	7.60±0.15 ^**C**,b^	5.69±0.15 ^**B**,d^	6.49±0.15 ^**B**,c^	4.53±0.15 ^**A**,c^
**MDE0**	5.70±0.15 ^**A**,b^	2.62±0.10 ^**B**,c^	7.81±0.18 ^**B**,a^	5.93±0.16 ^**C**,b^	6.49±0.13 ^**C**,a^	4.31±0.13 ^**C**,b^
**MDE1**	5.82±0.18 ^**A**,c^	2.73±0.11 ^**B**,b^	8.36±0.18 ^**B**,c^	5.61±0.16 ^**C**,c^	6.47±0.15 ^**C**,d^	5.27±0.14 ^**C**,d^
**MDE2**	5.93±0.17 ^**A**,d^	2.90±0.13 ^**B**,d^	12.56±0.19 ^**B**,d^	6.50±0.17 ^**C**,a^	7.72±0.16 ^**C**,b^	4.49±0.12 ^**C**,a^
**MDE3**	6.24±0.19 ^**A**,a^	2.48±0.15 ^**B**,a^	12.56±0.16 ^**B**,b^	6.11±0.16 ^**C**,d^	7.41±0.13 ^**C**,c^	4.43±0.11 ^**C**,c^
**HDE0**	6.46±0.17 ^**B**,b^	2.78±0.13 ^**A**,c^	8.54±0.16 ^**A**,a^	4.74±0.14 ^**A**,b^	5.54±0.14 ^**A**,a^	4.37±0.14 ^**B**,b^
**HDE1**	5.91±0.18 ^**B**,c^	2.42±0.14 ^**A**,b^	8.54±0.15 ^**A**,c^	5.54±0.16 ^**A**,c^	6.24±0.13 ^**A**,d^	4.54±0.14 ^**B**,d^
**HDE2**	5.74±0.16 ^**B**,d^	2.78±0.13 ^**A**,d^	8.84±0.15 ^**A**,d^	5.84±0.20 ^**A**,a^	6.34±0.21 ^**A**,b^	4.23±0.15 ^**B**,a^
**HDE3**	5.84±0.18 ^**B**,a^	2.62±0.12 ^**A**,a^	8.94±0.14 ^**A**,b^	5.64±0.15 ^**A**,d^	6.04±0.11 ^**A**,c^	5.26±0.16 ^**B**,c^

* Trt: Treatments; Significant differences (P < 0.05) among the three density treatments (LDE, MDE, HDE) are marked by bold, uppercase red superscripts, while differences between the four EDTA supplementation levels within each density treatment are indicated by lowercase superscripts.

#### 3.4.2. Monounsaturated fatty acids

A significant difference (P<0.05) was observed in tetrasenoic acid (df_2_, F = 319.08), pentadecenoic acid (df_2_, F = 54.25), palmitoleic acid (df_2_, F = 100581.25), oleic acid (df_2_, F = 21760.08) and heptadecenoic acid (df_2_, F = 179.08) between three density treatments Table 7. The effect of varying levels of EDTA (E) supplementation on tetrasenoic acid (df_3,_ F = 649.25), pentadecenoic acid (df_3,_ F = 82.44), palmitoleic acid (df_3,_ F = 5949.22), heptadecenoic acid (df_3,_ F = 205.91) and oleic acid (df_3,_ F = 49549.69) was noted to be significant (P<0.05) within each density treatment. The combined effect of density*EDTA concentration on tetrasenoic acid (df_6,_ F = 1156.75), pentadecenoic acid (df_6,_ F = 346.69), palmitoleic acid (df_6,_ F = 93484.08), heptadecenoic acid (df_6,_ F = 333.08) and oleic acid (df_6,_ F = 74223.52) was found to be significant (P<0.05) between three density treatments.

#### 3.4.3. Polyunsaturated fatty acids

In case of linoleic acid (df_2_, F = 2914.33) eicosadienoic acid (df_2_, F = 214.08), α-linolenic acid (df_2_, F = 258811.58), eicosapentanoic acid (df_2_, F = 25149.25), decosapentanoic acid (df_2_, F = 73116.75), and decosahexanoic acid (df_2_, F = 114.33), a significant difference (P<0.05) was observed between three density treatments Table 7. While the linoleic acid (df_3,_ F = 319.66), eicosadienoic acid (df_3,_ F = 1806.00), α-linolenic acid (df_3,_ F = 5438.80), eicosapentanoic acid (df_3,_ F = 724.88), decosapentanoicacid (df_3,_ F = 810.44) and decosahexanoic acid (df_3,_ F = 6551.02), the effect of different levels of EDTA supplementation was found to be significant (P<0.05) within each density treatment. The interactive effect of density*EDTA concentration on linoleic acid (df_6,_ F = 9618.00), eicosadienoic acid (df_6,_ F = 1581.41), α-linolenic acid (df_6,_ F = 490158.47), eicosapentanoic acid (df_6,_ F = 35044.13), decosapentanoic acid (df_6,_ F = 45815.19) and decosahexanoic acid (df_6,_ F = 12178.44), was also found to be significant (P<0.05) between three density treatments.

### 3.5. Digestive enzymes activity

**Fig 1:**
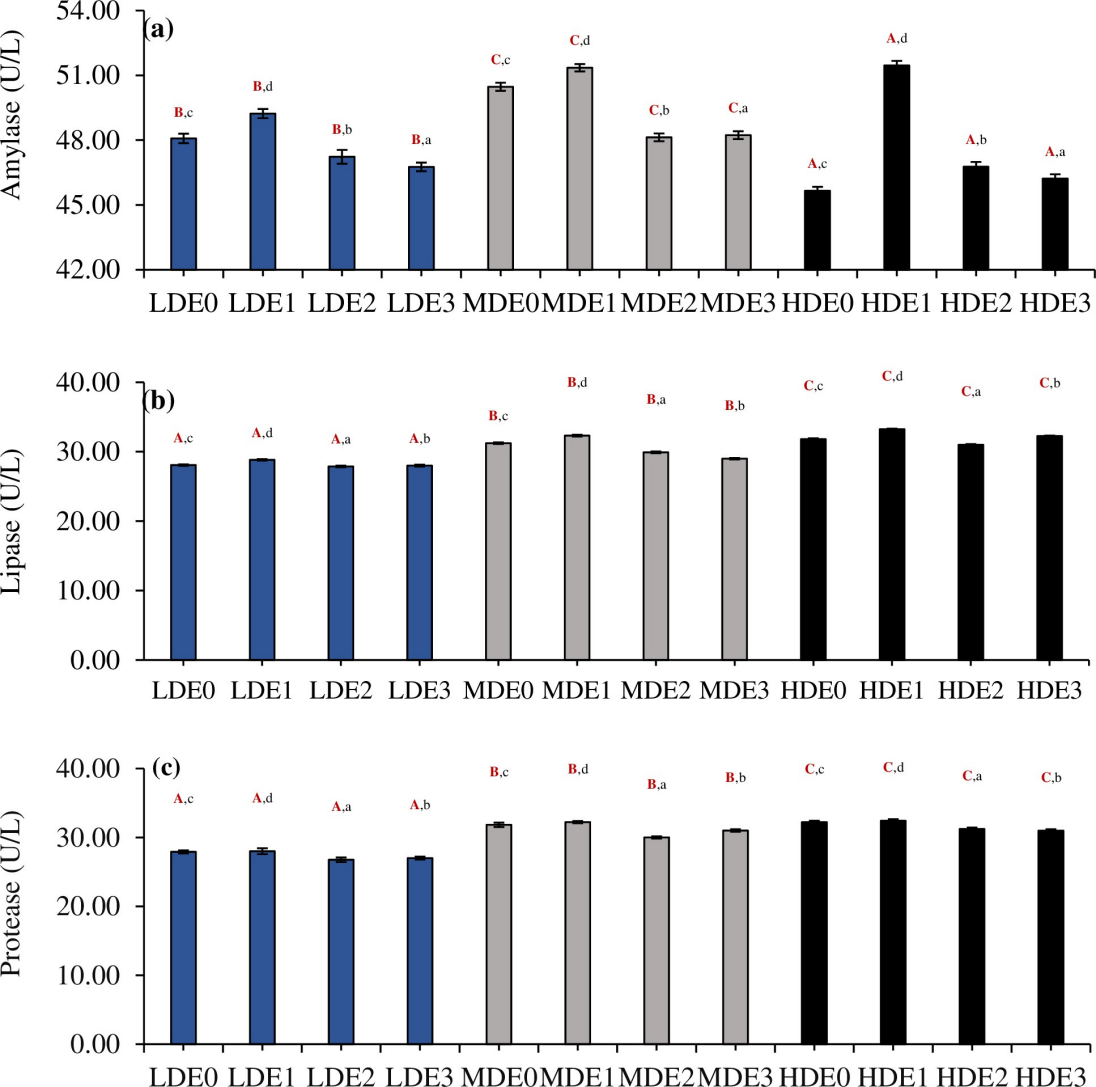
Three density treatments (LDE at 2.00 kg/m^3^, MDE at 3.50 kg/m^3^, HDE at 5.00 kg/m^3^) with four EDTA supplementation levels (E0 = 0 g/kg, E1 = 5 g/kg, E2 = 10 g/kg, and E3 = 15 g/kg) were used to assess the levels of (A) amylase, (B) lipase, and (C) protease (Mean ± SE). The three density treatments (LDE, MDE, and HDE) have significant differences (P < 0.05) indicated by superscripts in uppercase, bold, and red letters. Significant differences (P < 0.05) between the four EDTA supplementation levels within each density treatment are shown by superscripts in lowercase characters.

The activity of amylase (df_2_, F = 332677.33), protease (df_2_, F = 1562449.00) and lipase (df_2_, F = 1076335.75) was significantly different (P<0.05) between three density treatments (LDE, MDE, HDE) Fig 1. Different levels of EDTA supplementation within each density treatment (four in each treatment) also showed significant variations (P<0.05) in the activity of amylase (df_3_, F = 565899.44), lipase (df_3_, F = 150113.25) and protease (df_3_, F = 119403.47). In addition to this, the interactive effect of density*EDTA concentration on the activity of amylase (df_6_, F = 97912.11), lipase (df_6_, F = 35811.41) and protease (df_6_, F = 6121.88) was found to be significant (P<0.05) between three density treatments.

### 3.6. Profile of cortisol

**Fig 2:**
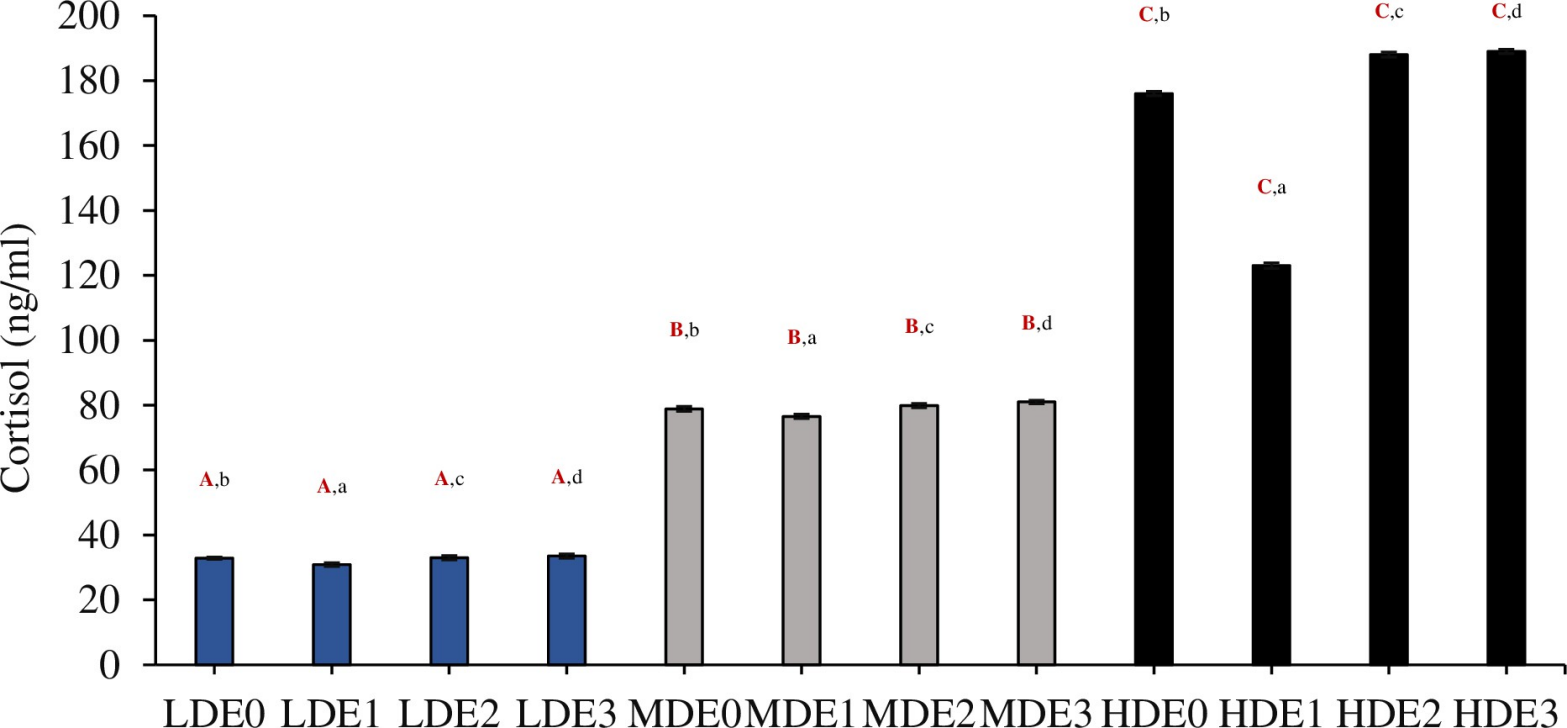
Three density treatments (LDE at 2.00 kg/m^3^, MDE at 3.50 kg/m^3^, and HDE at 5.00 kg/m^3^) were used to measure the cortisol level (Mean ± SE). Each treatment had four levels of EDTA supplementation (E0 = 0 g/kg, E1 = 5 g/kg, E2 = 10 g/kg, and E3 = 15 g/kg). The three density treatments (LDE, MDE, and HDE) have significant differences (P < 0.05) indicated by superscripts in uppercase, bold, and red letters. Significant differences (P < 0.05) between the four EDTA supplementation levels within each density treatment are shown by superscripts in lowercase characters.

The level of cortisol differed significantly (df_2_, F = 1346825025.00, P<0.05) between three density treatments (LDE, MDE, HDE) Fig 2. The effect of different levels of EDTA supplementation (four in each treatment) on cortisol within each treatment was noted to be significant (df_3_, F = 27263816.22, P<0.05). The interactive effect of density*EDTA concentration on the level of cortisol was also found to be significant (df_6_, F = 20677504.55, P<0.05) between three density treatments.

### 3.7. Blood biochemistry and hematology

#### 3.7.1. Hematology

A significant effect (P<0.05) was observed in the content of Hb (df_2_, F = 173658.33), platelets (df_2_, F = 52500.00), WBC (df_2_, F = 179802.58), RBC (df_2_, F = 830.08), HCT (df_2_, F = 7233.33), MCV (df_2_, F = 46223333.33), MCH (df_2_, F = 7180833.33), MCHC (df_2_, F = 11707500.00), monocytes (df_2_, F = 2858.33), eosinophils (df_2_, F = 408.33), neutrophils (df_2_, F = 1790833.33) and lymphocytes (df_2_, F = 1773333.33) between three density treatments Table 8.

**Table 8:** Three density treatments (LDE, MDE, and HDE) were used to analyze the blood hematological parameters (Mean ± SE). Each treatment had four levels of EDTA supplementation (E0 = 0 g/kg, E1 = 5 g/kg, E2 = 10 g/kg, and E3 = 15 g/kg).

**Trt**	**Hemoglobin (g/dl)**	**Total RBC (× 10** ^**6**^ **/μL)**	**HCT (%)**	**MCV (Fl)**	**MCH (pg)**	**MCHC (g/DL)**
**LDE0**	7.10±0.13 ^**C**,c^	1.07±0.06 ^**B**,d^	17.00±0.12 ^**A**,d^	145.00±0.99 ^**A**,a^	66.00±0.56 ^**B**,a^	45.00±0.65 ^**A**,b^
**LDE1**	7.30±0.14 ^**C**,d^	0.90±0.07 ^**B**,c^	18.00±0.23 ^**A**,c^	149.00±0.89 ^**A**,c^	69.00±0.76 ^**B**,b^	46.00±0.75 ^**A**,d^
**LDE2**	7.10±0.12 ^**C**,b^	0.78±0.05 ^**B**,a^	15.00±0.11 ^**A**,a^	165.00±0.78 ^**A**,d^	73.00±0.65 ^**B**,d^	34.00±0.45 ^**A**,c^
**LDE3**	7.00±0.11 ^**C**,a^	0.87±0.06 ^**B**,b^	17.00±0.21 ^**A**,b^	153.00±0.67 ^**A**,b^	75.00±0.57 ^**B**,c^	22.00±0.56 ^**A**,a^
**MDE0**	7.00±0.16 ^**B**,c^	1.08±0.09 ^**A**,d^	17.22±0.12 ^**C**,d^	166.00±0.87 ^**B**,a^	74.00±0.56 ^**C**,a^	44.00±0.54 ^**C**,b^
**MDE1**	7.20±0.17 ^**B**,d^	0.88±0.07 ^**A**,c^	18.11±0.21 ^**C**,c^	177.00±0.78 ^**B**,c^	76.00±0.55 ^**C**,b^	48.00±0.66 ^**C**,d^
**MDE2**	6.90±0.16 ^**B**,b^	0.78±0.09 ^**A**,a^	16.00±0.12 ^**C**,a^	181.00±0.78 ^**B**,d^	82.00±0.67 ^**C**,d^	52.00±0.56 ^**C**,c^
**MDE3**	7.10±0.19 ^**B**,a^	0.77±0.08 ^**A**,b^	16.87±0.12 ^**C**,b^	176.00±0.67 ^**B**,b^	80.00±0.76 ^**C**,c^	54.00±0.54 ^**C**,a^
**HDE0**	6.60±0.22 ^**A**,c^	1.09±0.09 ^**C**,d^	20.00±0.23 ^**B**,d^	184.00±0.87 ^**C**,a^	60.00±0.56 ^**A**,a^	32.00±0.67 ^**B**,b^
**HDE1**	6.90±0.23 ^**A**,d^	1.00±0.07 ^**C**,c^	17.00±0.12 ^**B**,c^	178.00±0.88 ^**C**,c^	74.00±0.56 ^**A**,b^	45.00±0.56 ^**B**,d^
**HDE2**	5.90±0.23 ^**A**,b^	0.86±0.06 ^**C**,a^	15.00±0.12 ^**B**,a^	171.00±0.98 ^**C**,d^	70.00±0.67 ^**A**,d^	47.00±0.54 ^**B**,c^
**HDE3**	3.50±0.27 ^**A**,a^	0.98±0.07 ^**C**,b^	16.00±0.14 ^**B**,b^	169.00±0.87 ^**C**,b^	69.00±0.56 ^**A**,c^	41.00±0.67 ^**B**,a^
**Trt**	**Platelets (× 10** ^**3**^ **/μL)**	**WBC (× 10** ^**3**^ **/μL)**	**Neutrophils (%)**	**Lymphocytes (%)**	**Monocytes (%)**	**Eosinophils (%)**
**LDE0**	144.00±0.55 ^**B**,d^	34.35±0.16 ^**B**,c^	86.00±0.39 ^**B**,d^	14.00±0.54 ^**B**,a^	2.00±0.01 ^**A**,a^	2.30±0.03 ^**C**,d^
**LDE1**	155.00±0.45 ^**B**,c^	33.00±0.18 ^**B**,d^	81.00±0.42 ^**B**,c^	19.00±0.53 ^**B**,b^	2.00±0.02 ^**A**,b^	2.30±0.03 ^**C**,c^
**LDE2**	145.00±0.43 ^**B**,b^	32.00±0.17 ^**B**,a^	83.00±0.40 ^**B**,b^	24.00±0.45 ^**B**,d^	2.00±0.03 ^**A**,b^	2.30±0.03 ^**C**,a^
**LDE3**	140.00±0.45 ^**B**,a^	32.54±0.18 ^**B**,b^	78.00±0.55 ^**B**,a^	17.00±0.53 ^**B**,c^	2.00±0.03 ^**A**,b^	2.40±0.02 ^**C**,b^
**MDE0**	154.00±0.55 ^**A**,d^	33.33±0.19 ^**C**,c^	88.00±0.51 ^**A**,d^	17.00±0.55 ^**C**,a^	2.00±0.04 ^**A**,a^	2.40±0.04 ^**A**,d^
**MDE1**	150.00±0.59 ^**A**,c^	33.88±0.21 ^**C**,d^	85.00±0.54 ^**A**,c^	16.00±0.65 ^**C**,b^	2.00±0.0 ^**A**,b^	2.30±0.06 ^**A**,c^
**MDE2**	143.00±0.69 ^**A**,b^	32.54±0.23 ^**C**,a^	82.00±0.65 ^**A**,b^	23.00±0.76 ^**C**,d^	2.00±0.04 ^**A**,b^	2.10±0.06 ^**A**,a^
**MDE3**	134.00±0.77 ^**A**,a^	32.77±0.31 ^**C**,b^	72.00±0.76 ^**A**,a^	26.00±0.85 ^**C**,c^	2.00±0.03 ^**A**,b^	2.00±0.08 ^**A**,b^
**HDE0**	165.00±0.70 ^**A**,d^	30.37±0.33 ^**A**,c^	84.00±0.67 ^**C**,d^	16.00±0.87 ^**A**,a^	2.10±0.09 ^**B**,a^	2.40±0.07 ^**B**,d^
**HDE1**	155.00±0.71 ^**A**,c^	32.76±0.36 ^**A**,d^	90.00±0.68 ^**C**,c^	14.00±0.8 ^**A**,b^	2.20±0.08 ^**B**,b^	2.20±0.06 ^**B**,c^
**HDE2**	132.00±0.73 ^**A**,b^	31.22±0.34 ^**A**,a^	86.00±0.69 ^**C**,b^	15.00±0.69 ^**A**,d^	2.20±0.07 ^**B**,b^	2.10±0.05 ^**B**,a^
**HDE3**	129.00±0.79 ^**A**,a^	32.33±0.35 ^**A**,b^	85.00±0.66 ^**C**,a^	17.00±0.78 ^**A**,c^	2.20±0.05 ^**B**,b^	2.30±0.05 ^**B**,b^

* Trt: Treatments; LD: low density; MD: medium density; HD: high density; E: CaNa_2_EDTA; RBC: red blood cell; WBC: white blood cell blood cell; HCT: hematocrit; MCV: mean corpuscular volume; MCH: mean corpuscular hemoglobin; MCHC: mean corpuscular hemoglobin concentration; Significant differences (P < 0.05) among the three density treatments (LDE, MDE, HDE) are marked by bold, uppercase red superscripts, while differences between the four EDTA supplementation levels within each density treatment are indicated by lowercase superscripts.

The effect of different levels of EDTA supplementation on Hb (df_3_, F = 63622.22), WBC (df_3_, F = 59362.13), RBC (df_3_, F = 2845.88), HCT (df_3_, F = 318402.00), MCV (df_3_, F = 2216666.66), MCH (df_3_, F = 3157777.77), MCHC (df_3_, F = 2450000.00), platelets (df_3_, F = 206033333.33), lymphocytes (df_3_, F = 1345555.55), monocytes (df_3_, F = 58.33), eosinophils (df_3_, F = 1613.88) and neutrophils (df_3_, F = 2535555.55) was found to be significant (P<0.05) within each density treatment.

A significant effect (P<0.05) of density*EDTA concentration was observed on the content of Hb (df_6_, F = 52480.55), WBC (df_6_, F = 59306.13), RBC (df_6_, F = 129.30), HCT (df_6_, F = 86235.33), MCV (df_6_, F = 4573333.33), MCH (df_6_, F = 678611.11), MCHC (df_6_, F = 5430833.33), platelets (df_6_, F = 4602500.00), neutrophils (df_6_, F = 1059722.22), monocytes (df_6_, F = 58.33), eosinophils (df_6_, F = 1030.55) and lymphocytes (df_6_, F = 808888.88) between three density treatments.

#### 3.7.2. Blood biochemistry

Content of triglycerides (df_2_, F = 939580833.33), ALT (df_2_, F = 335640396.00), AST (df_2_, F = 526685833.33), ALP (df_2_, F = 1975790833.33), albumin (df_2_, F = 85190.58), glucose (df_2_, F = 234103333.33), total protein (df_2_, F = 10208.33), bilirubin (df_2_, F = 343.58) and cholesterol (df_2_, F = 462.58) was significantly different (P<0.05) between three density treatments Table 9.

**Table 9:** Plasma samples were subjected to three density treatments (LDE, MDE, and HDE) with four EDTA supplementation levels (E0 = 0 g/kg, E1 = 5 g/kg, E2 = 10 g/kg, and E3 = 15 g/kg) before the blood biochemistry parameters were analyzed (Mean ± SE).

**Trt**	**Triglycerides (mg/dl)**	**Cholesterol (ug/ul)**	**ALT (U/L)**	**AST (U/L)**	**Alkaline Phosphate (U/L)**
**LDE0**	111.00±0.77 ^**A**,c^	0.45±0.23 ^**A**,d^	19.00±0.88 ^**A**,a^	20.00±0.66 ^**A**,b^	75.00±0.35 ^**A**,c^
**LDE1**	98.00±0.87 ^**A**,a^	0.44±0.12 ^**A**,a^	20.00±0.76 ^**A**,b^	22.00±0.62 ^**A**,a^	71.00±0.31 ^**A**,a^
**LDE2**	101.00±0.88 ^**A**,b^	0.45±0.32 ^**A**,b^	25.00±0.67 ^**A**,c^	31.00±0.65 ^**A**,c^	88.00±0.36 ^**A**,d^
**LDE3**	121.00±0.89 ^**A**,d^	0.46±0.32 ^**A**,c^	30.00±0.81 ^**A**,d^	41.00±0.76 ^**A**,d^	92.00±0.44 ^**A**,b^
**MDE0**	121.00±0.99 ^**B**,c^	0.48±0.12 ^**B**,d^	26.00±0.80 ^**B**,a^	49.00±0.66 ^**B**,b^	99.00±0.48 ^**B**,c^
**MDE1**	111.00±0.88 ^**B**,a^	0.46±0.32 ^**B**,a^	26.44±0.78 ^**B**,b^	43.00±0.76 ^**B**,a^	109.00±0.55 ^**B**,a^
**MDE2**	121.00±0.98 ^**B**,b^	0.47±0.22 ^**B**,b^	29.00±0.85 ^**B**,c^	54.00±0.70 ^**B**,c^	145.00±0.76 ^**B**,d^
**MDE3**	133.00±0.88 ^**B**,d^	0.46±0.23 ^**B**,c^	32.00±0.81 ^**B**,d^	65.00±0.67 ^**B**,d^	122.00±0.67 ^**B**,b^
**HDE0**	226.00±0.77 ^**C**,c^	0.59±0.33 ^**C**,d^	67.00±0.77 ^**C**,a^	87.00±0.76 ^**C**,b^	288.00±0.76 ^**C**,c^
**HDE1**	177.00±0.80 ^**C**,a^	0.47±0.23 ^**C**,a^	66.00±0.74 ^**C**,b^	90.00±0.73 ^**C**,a^	201.00±0.78 ^**C**,a^
**HDE2**	199.00±0.86 ^**C**,b^	0.51±0.32 ^**C**,b^	79.00±0.73 ^**C**,c^	131.00±0.76 ^**C**,c^	245.00±0.76 ^**C**,d^
**HDE3**	225.00±0.81 ^**C**,d^	0.54±0.21 ^**C**,c^	131.00±0.74 ^**C**,d^	143.00±0.73 ^**C**,d^	234.00±0.70 ^**C**,b^
**Trt**	**Albumin (g/dl)**	**Bilirubin (mg/dl)**	**Total protein (g/dl)**	**Glucose (mg/dl)**	
**LDE0**	0.90±0.01 ^**A**,a^	2.10±0.09 ^**A**,b^	36.00±0.32 ^**C**,b^	72.00±0.87 ^**A**,b^	
**LDE1**	1.00±0.03 ^**A**,b^	1.90±0.03 ^**A**,a^	37.00±0.34 ^**C**,a^	73.00±0.89 ^**A**,a^	
**LDE2**	0.90±0.02 ^**A**,b^	2.00±0.05 ^**A**,b^	36.00±0.23 ^**C**,c^	76.00±0.78 ^**A**,c^	
**LDE3**	1.80±0.07 ^**A**,c^	2.40±0.06 ^**A**,c^	35.00±0.43 ^**C**,c^	88.00±0.79 ^**A**,d^	
**MDE0**	1.20±0.08 ^**B**,a^	2.40±0.07 ^**B**,b^	35.00±0.24 ^**B**,b^	80.00±0.87 ^**B**,b^	
**MDE1**	2.10±0.09 ^**B**,b^	2.40±0.06 ^**B**,a^	32.00±0.34 ^**B**,a^	79.00±0.85 ^**B**,a^	
**MDE2**	1.99±0.07 ^**B**,b^	2.50±0.04 ^**B**,b^	36.00±0.23 ^**B**,c^	101.00±0.76 ^**B**,c^	
**MDE3**	1.80±0.09 ^**B**,c^	2.60±0.04 ^**B**,c^	36.00±0.34 ^**B**,c^	111.00±0.70 ^**B**,d^	
**HDE0**	1.80±0.04 ^**C**,a^	2.70±0.03 ^**C**,b^	32.00±0.23 ^**A**,b^	112.00±0.74 ^**C**,b^	
**HDE1**	2.20±0.05 ^**C**,b^	2.60±0.05 ^**C**,a^	32.00±0.21 ^**A**,a^	101.00±0.73 ^**C**,a^	
**HDE2**	2.40±0.06 ^**C**,b^	2.90±0.07 ^**C**,b^	32.00±0.34 ^**A**,c^	155.00±0.72 ^**C**,c^	
**HDE3**	2.60±0.08 ^**C**,c^	3.00±0.09 ^**C**,c^	33.00±0.23 ^**A**,c^	165.00±0.87 ^**C**,d^	

* Trt: Treatments; LD: low density; MD: medium density; HD: high density; E: CaNa_2_EDTA; Significant differences (P < 0.05) among the three density treatments (LDE, MDE, HDE) are marked by bold, uppercase red superscripts, while differences between the four EDTA supplementation levels within each density treatment are indicated by lowercase superscripts.

The effect of different levels of EDTA supplementation on the level of triglycerides (df_3_, F = 64656666.66), glucose (df_3_, F = 66754722.22), ALT (df_3_, F = 34056596.00), AST (df_3_, F = 50353333.33), ALP (df_3_, F = 42410277.77), albumin (df_3_, F = 21039.47), total protein (df_3_, F = 466.66), bilirubin (df_3_, F = 50.36) and cholesterol (df_3_, F = 91.00), was found to be significant (P<0.05) within each density treatment.

The combined effect of density*EDTA concentration on triglycerides (df_6_, F = 14204166.66), glucose (df_6_, F = 11985555.55), ALT (df_6_, F = 17209462.66), AST (df_6_, F = 9432500.00), ALP (df_6_, F = 41325277.77), albumin (df_6_, F = 5849.47), total protein (df_6_, F = 1341.66), bilirubin (df_6_, F = 6.02) and cholesterol (df_6_, F = 49.58) was also noted to be significant (P<0.05) between three density treatments.

### 3.8. Antioxidant assay

A significant difference (P<0.05) was observed in the levels of CAT (df_2_, F = 468517.00), SOD (df_2_, F = 49147.58), GPx (df_2_, F = 187111.75) and MDA (df_2_, F = 91383.25) between three density treatments Table 10. A significant variation (P<0.05) in the levels of CAT (df_3_, F = 19617.11), SOD (df_3_, F = 7607.25), GPx (df_3_, F = 29028.80) and MDA (df_3_, F = 5595.91) was observed across different levels of EDTA supplementation (four in each treatment) within each treatment. The combined effect of density*EDTA concentration on the levels of CAT (df_6_, F = 51259.44), SOD (df_6_, F = 22862.58), GPx (df_6_, F = 65147.63) and MDA (df_6_, F = 4839.91) was also found to be significant (P<0.05) between three density treatments.

**Table 10:** The assay of antioxidant biomarkers (Mean ± SE) was conducted from plasma samples from three density treatments (LDE, MDE, HDE), each with four EDTA supplementation levels (E0 = 0 g/kg, E1 = 5 g/kg, E2 = 10 g/kg, E3 = 15 g/kg). These biomarkers included catalase (U/ml), superoxide dismutase (ng/ml), glutathione peroxidase (IU/ml), and malondialdehyde (nmol/ml).

Trt	Catalase (U/ml)	Superoxide dismutase (ng/ml)	Glutathione peroxidase (IU/ml)	Malondialdehyde (nmol/ml)
**LDE0**	13.90±0.10 ^**A**,d^	2.05±0.08 ^**A**,c^	24.80±0.21 ^**B**,c^	0.26±0.02 ^**A**,b^
**LDE1**	15.00±0.11 ^**A**,c^	2.20±0.07 ^**A**,d^	25.44±0.22 ^**B**,d^	0.18±0.06 ^**A**,a^
**LDE2**	13.22±0.12 ^**A**,a^	1.90±0.06 ^**A**,b^	24.00±0.26 ^**B**,b^	0.20±0.07 ^**A**,c^
**LDE3**	14.00±0.16 ^**A**,b^	0.99±0.08 ^**A**,a^	23.00±0.33 ^**B**,a^	0.23±0.06 ^**A**,d^
**MDE0**	14.90±0.17 ^**B**,d^ III	2.91±0.09 ^**C**,c^	25.01±0.32 ^**C**,c^	0.33±0.05 ^**B**,b^
**MDE1**	15.50±0.15 ^**B**,c^	3.10±0.06 ^**C**,d^	25.77±0.31 ^**C**,d^	0.29±0.06 ^**B**,a^
**MDE2**	14.33±0.13 ^**B**,a^	2.12±0.08 ^**C**,b^	23.88±0.28 ^**C**,b^	0.30±0.05 ^**B**,c^
**MDE3**	14.01±0.15 ^**B**,b^	2.22±0.08 ^**C**,a^	24.99±0.26 ^**C**,a^	0.32±0.06 ^**B**,d^
**HDE0**	17.30±0.17 ^**C**,d^	2.11±0.07 ^**B**,c^	23.54±0.28 ^**A**,c^	1.10±0.04 ^**C**,b^
**HDE1**	15.30±0.16 ^**C**,c^	1.88±0.08 ^**B**,d^	22.32±0.25 ^**A**,d^	0.70±0.07 ^**C**,a^
**HDE2**	16.50±0.15 ^**C**,a^	2.87±0.09 ^**B**,b^	24.11±0.27 ^**A**,b^	1.40±0.05 ^**C**,c^
**HDE3**	17.00±0.14 ^**C**,b^	2.72±0.08 ^**B**,a^	23.21±0.33 ^**A**,a^	1.80±0.06 ^**C**,d^

* Trt: Treatments; LD: low density; MD: medium density; HD: high density; E: CaNa_2_EDTA; Significant differences (P < 0.05) among the three density treatments (LDE, MDE, HDE) are marked by bold, uppercase red superscripts, while differences between the four EDTA supplementation levels within each density treatment are indicated by lowercase superscripts.

### 3.9. Gene expression

**Fig 3:**
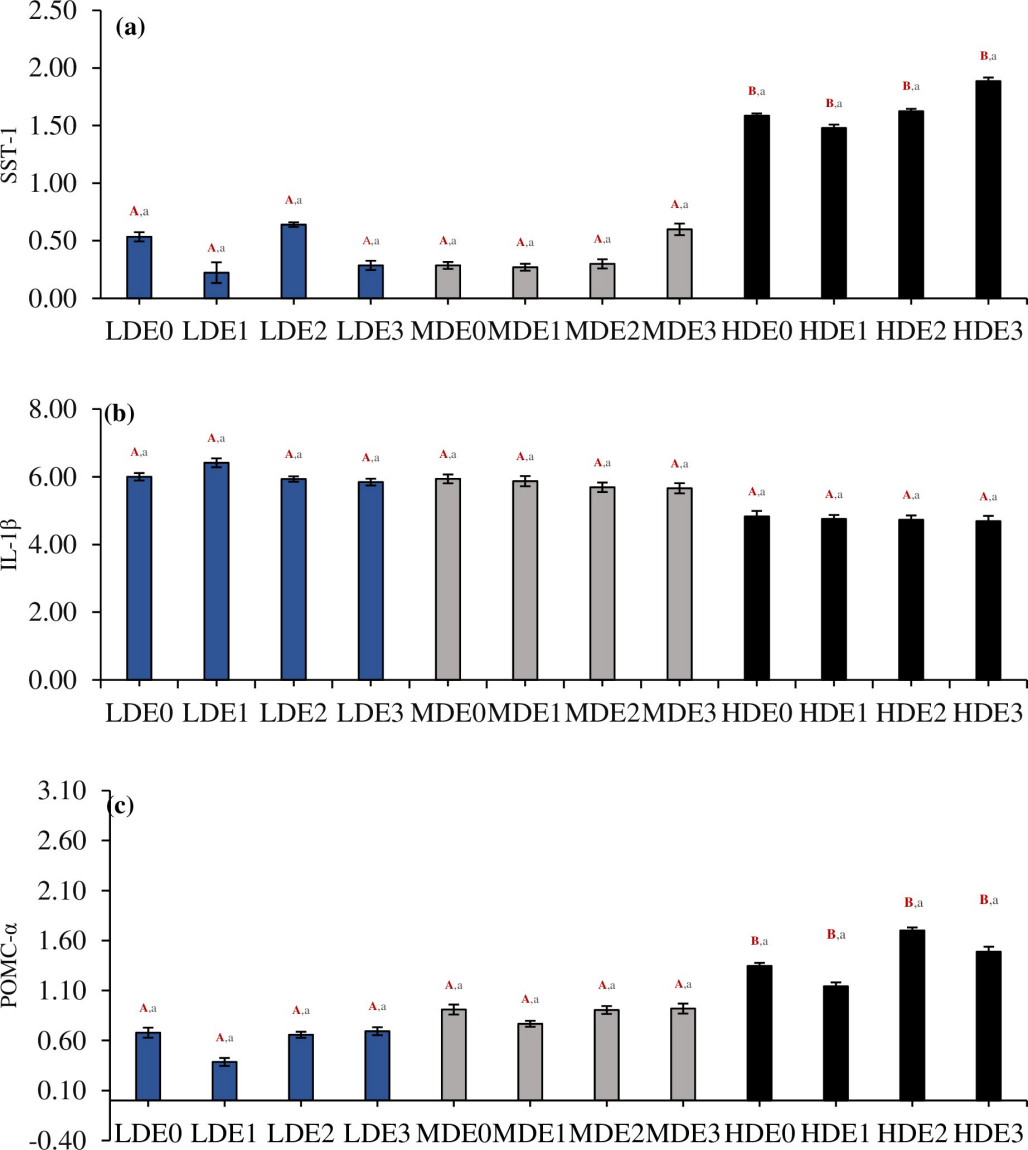
Four EDTA supplementation doses (E0 = 0 g/kg, E1 = 5 g/kg, E2 = 10 g/kg, and E3 = 15 g/kg) were present in each of the three density treatments (LDE: 2.00 kg/m^3^, MDE: 3.50 kg/m^3^, HDE: 5.00 kg/m^3^) where the levels of gene expression for (A) Somatostatin 1, (B) Interleukin 1-β, and (C) POMC-α (Mean ± SE) were determined. The three density treatments (LDE, MDE, and HDE) have significant differences (P < 0.05) indicated by superscripts in uppercase, bold, and red letters. Significant differences (P < 0.05) between the four EDTA supplementation levels within each density treatment are shown by superscripts in lowercase characters.

The expression of SST-1 gene (df_2_, F = 39.59) and POMC-α (df_2_, F = 13.24) was significantly different (P<0.05) between three density treatments. However, the expression of interleukin 1-β was insignificantly different (df_2_, F = 0.11, P>0.05) between three density treatments Fig 3. Different levels of EDTA supplementation within each treatment showed an insignificant effect (P>0.05) on the levels of SST-1 (df_3_, F = 0.73), interleukin 1-β (df_3_, F = 0.003) and POMC-α (df_3_, F = 1.16). The combined effect of density*EDTA concentration on the expression of SST-1 (df_6_, F = 0.52), interleukin 1-β (df_6_, F = 0.19) and POMC-α (df_6_, F = 0.21) was noted to be insignificant (P>0.05) between three density treatments.

### 3.10. Histological analysis

**Fig 4:**
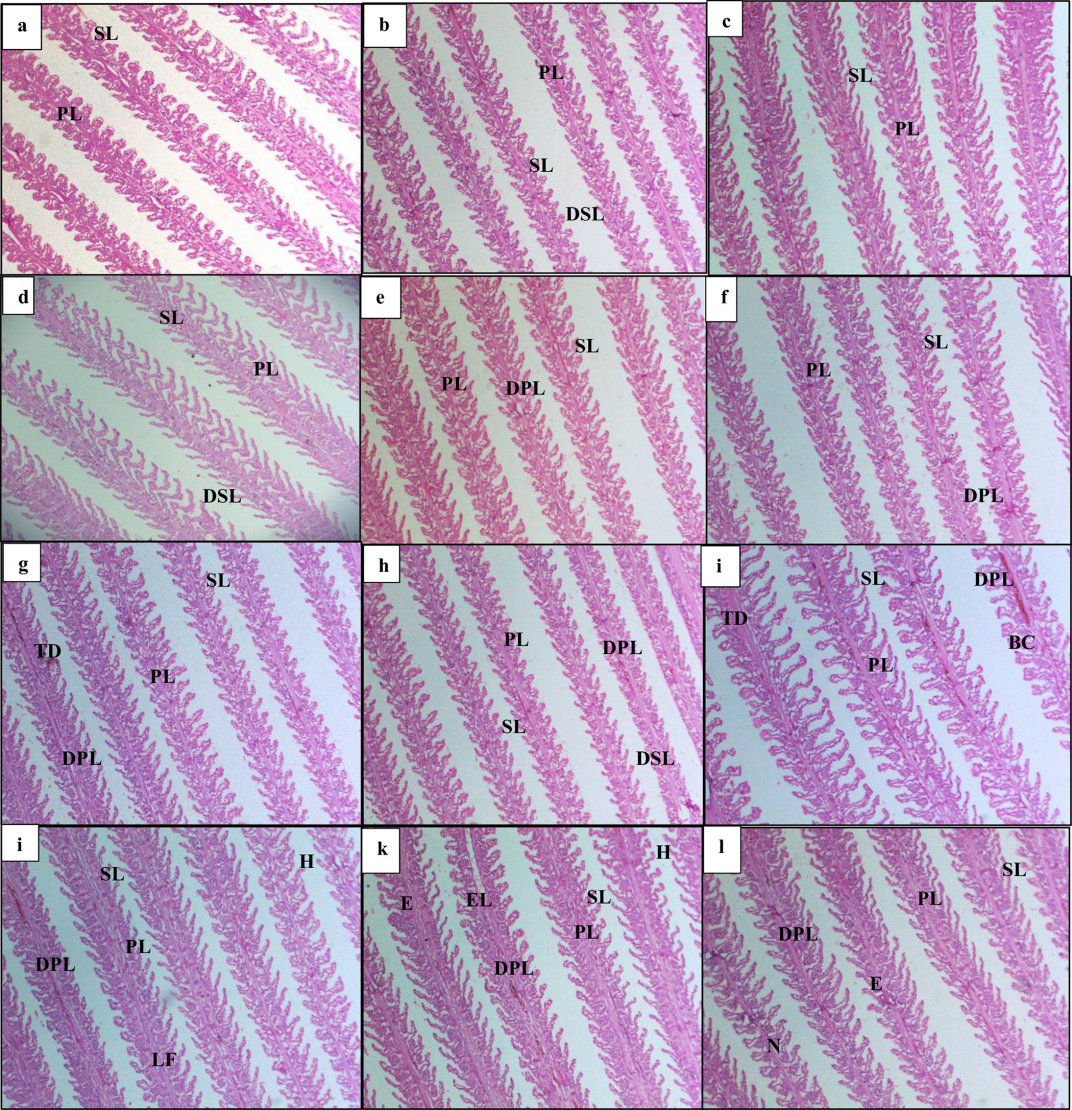
Histological changes in gills. Light micrographs of a paraffin section stained with eosin (10x). A(LDEO), B(LDE1), C(LDE2), D(LDE3), E(MDE0), F(MDE1), G(MDE2), H(MDE3), I(HDEO), J(HDE1), K(HDE2), L(HDE3). PL: Primary lamellae; SL: Secondary lamellae; DPL: Degeneration of primary lamellae; DSL: Degeneration of secondary lamellae; TD: Tissue debris; BC: Blood congestion; N: necrosis; EL: epithelial lifting; LF: Lamellar fusion; E: Edema; H: Hyperplasia.

Histological examination of the gills was conducted across all treatment groups (density * EDTA) Table 11, Fig 4. In the low-density treatment, there was only a slight disruption observed in the structure of the lamellae Fig 4B and 4D. In contrast, both the medium and high-density treatments exhibited significant alterations in gill structure. These changes were characterized by the degeneration of primary and secondary lamellae, along with the presence of tissue debris as illustrated in Fig 4E–4L, which was markedly more pronounced compared to the low-density treatment.

**Table 11:** Histopathological scoring of tilapia (*Oreochromis niloticus*) gills in three density treatments (LDE, MDE, HDE) with each treatment having four levels of EDTA supplementation (E0 = 0 g/kg, E1 = 5 g/kg, E2 = 10 g/kg, E3 = 15 g/kg).

Treatments	Degeneration of primary lamella	Degeneration of secondary lamella	Tissue debris	Lamellar fusion	Blood congestion	Edema	Necrosis	Hyperplasia
**LDE0**	0	0	0	0	0	0	0	0
**LDE1**	0	1	0	0	0	0	0	0
**LDE2**	0	0	0	0	0	0	0	0
**LDE3**	0	1	0	0	0	0	0	0
**MDE0**	1	0	0	0	0	0	0	0
**MDE1**	1	0	0	0	0	0	0	0
**MDE2**	1	0	1	0	0	0	0	0
**MDE3**	1	1	0	0	0	0	0	0
**HDE0**	3	0	3	0	3	0	0	0
**HDE1**	3	1	0	3	0	0	0	3
**HDE2**	3	0	0	3	1	1	0	3
**HDE3**	3	0	0	3	0	0	3	0

*0: Normal; 1: Mild alteration; 3: High alterations

Specifically, the high-density treatment displayed several pathological changes, including lamellar fusion Fig 4J, which indicates an abnormal merging of the lamellae. Additionally, signs of necrosis were evident Fig 4L, along with epithelial lifting Fig 4K, where epithelial cells were detached from the secondary lamellae. Furthermore, congestion of blood vessels within the gills was also observed Fig 4I, indicating impaired blood flow and potentially exacerbating the overall stress experienced by the fish in high-density conditions. Fusion of lamella structure has been observed in gills structure in all three density treatments. While, edema which is the abnormal accumulation of fluid in interstitial space or in tissues was observed Fig 4K and 4I. Hyperplasia which is increase in cell or tissue enlargement has also been observed Fig 4I. Low density treatment showed normal structure of gills including primary lamella and secondary lamella with less or no structural alterations.

## 4. Discussion

Numerous studies have demonstrated that crowding stress, in the absence of beneficial dietary supplements, adversely impacts fish health and overall well-being [53, 54]. This study is the first to explore the potential role of EDTA supplementation in mitigating oxidative stress induced by varying stocking densities. The results revealed that dietary supplementation with EDTA (E1 = 5 g/kg) marginally enhanced growth parameters in fish reared under high-density conditions (HD = 5.00 kg/m^3^). These findings are consistent with previous research conducted on species such as beluga (*Huso huso*) [55] and tilapia [56, 57]. However, there is limited literature on the effects of EDTA on the growth of aquatic species, necessitating further investigation to better understand its potential benefits. The observed weight gain following EDTA supplementation may be attributed to enhanced digestion and nutrient absorption in fish exposed to stressors [58], similar to the effects of acidifiers in fish diets [58, 59]. Acidifiers have been shown to improve gut function and stimulate digestive enzymes by lowering the pH of the gastrointestinal tract [60]. Additionally, EDTA’s strong chelating properties facilitate the removal of intestinal impurities, potentially enhancing mineral absorption and bioavailability [56]. Furthermore, fish growth performance has been linked to the somatostatin gene-1 (SST-I). This study observed increased SST-I gene expression under high stocking density conditions. However, following the dietary supplementation of EDTA (E1 = 5 g/kg), SST-I expression decreased, suggesting a positive effect on growth. Notably, previous research has not yet explored the influence of EDTA supplementation on SST-I gene expression. Proteins are fundamental to body structure, comprising a substantial portion of cellular matter and accounting for nearly half of a cell’s dry weight [61]. They are vital for regulating various physiological and metabolic processes within the body [62]. The present study demonstrates that, in addition to growth parameters, the chemical composition particularly crude protein showed a slight increase with EDTA dietary supplementation across different stocking densities. Statistically, significant differences were observed in the chemical composition of fish, including protein, fat, ash content, and the profiles of amino acids and fatty acids, although the quantitative effects were not pronounced. These findings are consistent with previous studies conducted on *Oreochromis niloticus*, *Sarotherodon galilaeus*, and *Heteropneustes fossilis* [56, 57, 63]. Furthermore, a significant enhancement in the activity of digestive enzymes, specifically amylase, lipase, and protease, was observed. This suggests that dietary EDTA supplementation may positively impact digestive and absorptive functions by removing impurities from the intestines, thus improving the digestion and absorption of nutrients. These results indicate that EDTA, as a potent chelator, can enhance mineral uptake and bioavailability in fish diets, similar to effects previously documented in poultry [64, 65]. The present study observed a reduction in hemoglobin levels in fish reared at high density following dietary supplementation with EDTA, compared to the E0 treatment. Hemoglobin, along with red blood cells (RBCs), plays a vital role in transporting oxygen to tissues and facilitating the removal of harmful substances through the gills [66]. Interestingly, a previous study on beluga (*Huso huso*) reported no effect on blood hematology with EDTA supplementation [55]. In addition, this study found elevated triglyceride levels in fish reared under high-density conditions compared to those in low and medium densities. However, with EDTA supplementation, triglyceride levels decreased, possibly due to improved lipid metabolism in tilapia, potentially through enhanced lipolysis and reduced fat accumulation [67]. Furthermore, the study observed an increase in white blood cell (WBC) count with EDTA supplementation in high-density environments compared to the E0 treatment, indicating a potential enhancement of the immune response in fish under these conditions. Under high-density conditions, the highest levels of ALT, AST, and ALP enzymes were observed in the E0 treatment, indicating potential liver cell damage. These enzymes are normally contained within cells but are released into the bloodstream when cellular integrity is compromised [68]. However, in EDTA-supplemented treatments, the levels of these enzymes decreased, as previously noted in tilapia [56, 57]. The present study also recorded elevated cortisol and glucose levels in high-density treatments compared to low and medium densities. However, supplementation with the E1 dose significantly reduced both cortisol and glucose levels in high-density conditions relative to the E0 treatment, consistent with prior findings in *Oreochromis niloticus* [56], where glucose levels decreased following EDTA supplementation. The mechanism behind the reduction in cortisol levels with E1 treatment in high-density conditions requires further investigation. Additionally, the study assessed stress at the molecular level by measuring POMC-α expression. No significant differences were found in POMC-α expression across density and supplementation treatments. Elevated POMC-α expression is typically linked to increased stress, as it triggers activation of the hypothalamic-pituitary-adrenal (HPA) axis [69, 70]. This activation initiates the release of corticotropin-releasing factor, which promotes the synthesis of pituitary pro-opiomelanocortin (POMC) [71, 72]. POMC is subsequently processed into adrenocorticotropic hormone (ACTH), which stimulates cortisol release through the melanocortin 2 receptor [71–73]. The current study highlights an increase in oxidative enzyme activity (CAT, SOD, GPx) under high-density conditions in fish fed the E0 diet. In contrast, a reduction in these enzymes was observed with dietary EDTA supplementation, particularly with the E1 dose under HDE1 treatment. This decline in antioxidant enzyme activity may be linked to EDTA’s well-established role as a potent antioxidant [74], which significantly reduces free radical production [44]. Similar results have been reported in *Oreochromis niloticus* regarding SOD and GPx activity [56]. SOD, GPx, and CAT are crucial antioxidant enzymes [75] that play a key role in neutralizing superoxide (O_2_−), hydrogen peroxide (H_2_O_2_), and lipid hydroperoxides (ROOH) generated by free radicals. For example, SOD catalyzes the dismutation of superoxide radicals: O_2_− + O_2_− + 2H+ → H_2_O_2_ + O_2_. The resulting hydrogen peroxide is then broken down by GPx and CAT [75]. Additionally, malondialdehyde (MDA), a marker of oxidative stress related to lipid peroxidation [76], typically increases under oxidative stress. However, in contrast to previous studies, this research found a decrease in MDA levels following EDTA supplementation [56].

Fish health is influenced by both their antioxidant defense mechanisms and immune responses, which collectively ensure their well-being. In this study, the immune response was assessed at the molecular level by examining the expression of the IL-1β gene. IL-1β, produced by activated macrophages, is critical in modulating innate immunity and inflammatory responses [77]. A significant variation in IL-1β expression was observed in response to both stocking density and dietary supplementation treatments. To our knowledge, no previous studies have specifically investigated IL-1β expression in tilapia in relation to EDTA supplementation, making this finding unique.

## 5. Conclusion

This study demonstrated that dietary supplementation with EDTA enhanced growth and antioxidant responses, effectively reducing oxidative stress in high-density stocking conditions. The E1 dose (5 g/kg) was identified as the most effective in alleviating stress parameters, particularly in the high-density group (5.00 kg/m^3^). However, EDTA did not significantly impact stress markers or immune function at the molecular level, as indicated by unchanged POMC-α and IL-1β expression. These findings suggest that EDTA, particularly at the E1 dose, can be a valuable addition to intensive aquaculture systems to improve fish health and productivity under high stocking densities.

### 5.1. Recommendations

The administration of several others nutraceuticals at optimal dosages will help mitigate stress associated with high stocking densities by reducing oxidative stress through the upregulation of antioxidant biomarkers and enhanced free radical scavenging activity. Future studies should explore alternative dosing regimens beyond those used in this research to further optimize oxidative stress mitigation. It will be crucial for the dietary supplements to remain cost-effective to ensure their adoption by farmers. This approach will enable higher stocking densities in intensive aquaculture systems, leading to increased production yields, meeting nutritional demands, and positively impacting food security and the economy.

## Supporting information

S1 Data (XLSX)
